# Biological heart and brain ageing in subjects with cardiovascular diseases

**DOI:** 10.3389/fcvm.2025.1569423

**Published:** 2025-07-07

**Authors:** Elizabeth Mcavoy, Matthias Wilms, Nils D. Forkert

**Affiliations:** ^1^ Department of Radiology, University of Calgary, Calgary, AB, Canada; ^2^ Hotchkiss Brain Institute, University of Calgary, Calgary, AB, Canada; ^3^ Department of Biomedical Engineering, University of Calgary, Calgary, AB, Canada; ^4^ Alberta Children’s Hospital Research Institute, University of Calgary, Calgary, AB, Canada; ^5^ Department of Pediatrics, University of Calgary, Calgary, AB, Canada; ^6^ Department of Community Health Sciences, University of Calgary, Calgary, AB, Canada; ^7^ Department of Clinical Neuroscience, University of Calgary, Calgary, AB, Canada

**Keywords:** brain age gap, heart age gap, cardiovascular disease, ageing, heart-brain axis

## Abstract

**Introduction:**

The heart-brain axis hypothesis suggests a bidirectional connection between the brain and the heart with relevant implications in health and disease. Cardiovascular diseases have been empirically linked to an increased risk of neurological diseases. However, it remains unclear to what extent different cardiovascular diseases affect brain health quantitatively across subjects and if that is associated with the extent the heart is affected by a disease. Therefore, this study aims to explore how cardiovascular diseases affect biological ageing of the brain and heart by quantifying the brain age gap (BAG) and the heart age gap (HAG) and relating the two to each other.

**Methods:**

This study used data from UK Biobank participants with available T1-weighted brain magnetic resonance imaging (MRI) scans, cardiac MRI-derived features, as well as pulse wave analysis cardiac measurements. This dataset included 7,500 healthy females and 6,684 healthy males. The data from healthy subjects was used to train biological brain age prediction machine learning models. For BAG computation, a convolutional neural network was trained based on the MRI data, while a CatBoost model was trained for HAG analyses based on the tabulated cardiac features. Individuals with cardiovascular diseases (F = 2,304, M = 2,925) in the UK Biobank were categorized using Phecodes and split based on sex and used to calculate the HAG and BAG for further analyses.

**Results:**

In 36 sex-specific cardiovascular disease groups, 24 showed significant differences from healthy subjects in the BAG and HAG distributions, whereas no strong correlations between the BAG and HAG distributions within disease groups were found. However, some diseases, such as hypotension and cardiac conduction disorders, showed sex-specific differences.

**Discussion:**

This study demonstrates that the combined use of HAG and BAG biomarkers provides a more comprehensive understanding of the interplay between cardiovascular and neurological ageing. The significant differences observed in disease groups, while lacking a strong correlation between the BAG and HAG, questions the generalizability of the heart-brain axis theory with respect to age gap biomarkers, suggesting potentially heterogeneous aging processes of the two systems that warrant further investigation in future work.

## 1 Introduction

A growing body of recent research supports the theory of the so-called heart-brain axis (1), which posits that there is a bidirectional connection between the brain and the heart so that diseases in one organ may also manifest as dysfunctions in the other. For example, reciprocal links between Alzheimer's disease and heart failure have been reported in the literature, with the diagnosis of one leading to an increased risk of development of the other (2, 3). Other studies have suggested a bidirectional relationship between cardiac disease and brain volume changes (4). This bidirectional link may be related to the close physiological and pathological connections between the heart and brain.

For many diseases, including cardiac and neurological diseases, the chronological age of a person is an important risk factor (5). However, the chronological age of a person may not be an optimal risk assessment tool due to considerable inter-individual differences in biological ageing, which can be related to environmental, lifestyle, genetic, and many other factors. Within this context, the biological age refers to how the body or organ system appears from an anatomical or functional perspective when compared to a healthy/typical ageing trajectory. From a technical point of view, the biological age of an organ can be estimated using a machine learning model that was trained using imaging or other clinical data from healthy subjects for whom it is assumed that the biological age is equal to their chronological age. Particularly, when applied to data from presumed non-healthy subjects, it can be used to calculate the organ-specific age gap biomarker, which is defined by the difference between the biological age and chronological age. Various age gap biomarkers have been proposed in the literature so far, including the brain age gap (BAG), which has been shown to be a sensitive biomarker for many neurological diseases (6), the retinal age gap (7), which has shown promise for the detection of ocular and some non-ocular diseases (8), and the heart age gap (HAG), which has been used to quantify deviations from healthy ageing in subjects with cardiac diseases (9, 10).

The brain age gap was one of the first imaging biomarkers that was suggested in this realm and is typically computed using 3D anatomical magnetic resonance imaging (MRI) of the brain using convolutional neural networks (11–13). With relevance to the heart-brain axis assumption, recent research has, for example, shown that subjects with an increased risk of cardiovascular diseases (14–16) or those diagnosed with cardiovascular diseases (12) exhibit significant deviations in brain age gaps compared to healthy controls. However, none of these previous studies took into account that subjects with cardiac diseases differ with respect to how various cardiac diseases manifest clinically, and it remains unclear whether there is a clear link between biological heart and brain ageing in subjects with specific cardiac diseases.

More recently, biological cardiovascular ageing has started to be investigated using similar approaches as commonly used in biological brain age research, whereas deviations in structural and functional features of the heart are now used to compute the biological heart age biomarker. In the heart, the general/typical ageing process manifests through alterations in tissue structure, such as fibrosis, arterial stiffening, and changes in myocardial function (17, 18). Therefore, the HAG has been investigated using electrocardiograms (ECG) (19–23), echocardiograms (24), and cardiac MRI (25–27). Within this context, it has been found that the ECG-derived heart age gap is correlated with heart age gaps computed using cardiac MRI data (28, 29), and that the combination of ECG data and MRI does not improve age prediction performance (25). From a technical point of view, the HAG can be used as a surrogate measure to quantify the severity of the effect of a cardiovascular disease on the heart and its ageing process. However, to the best of our knowledge, the HAG and BAG have not been investigated in combination for a wide range of specific cardiac diseases so far as a means to explore a potential heart-brain axis in these diseases.

Thus, this work aimed to build on previous HAG and BAG investigations but now with a focus on analyzing to what extent cardiovascular diseases are associated with changes in the HAG and BAG and if there is any relevant correlation between the two biomarkers that provides additional experimental support for the heart-brain axis hypothesis.

## 2 Materials and methods

### 2.1 Data

This research has been conducted using the UK Biobank Resource (30) under Application Number 77508. This data was used for this work as it provides detailed data for a large number of healthy participants and individuals with various diseases. This data includes T1-weighted MRI of the brain, as well as derived cardiac MRI (31) and measured pulse wave analysis features.

The study population available was divided into two primary groups, healthy individuals (*n* = 14,184) and those with cardiovascular diseases (*n* = 5,229). This was done by following the procedures and exclusion criteria outlined in McAvoy et al. (32), whereas patients with diseases not under investigation that have previously been shown to be associated with deviations in BAG were excluded. This included neurological and mental diseases, as well as diseases such as diabetes, chronic kidney disease, and human immunodeficiency virus. Additionally, patients with diseases that could greatly affect the morphology of the brain or heart were excluded, such as cancers of the heart and brain, stroke, and traumatic injuries of the heart and brain. The final healthy cohort available and used for this work consisted of 7,500 female and 6,684 male participants. All data groups were split based on sex for all analyses, as both the brain and the heart have been shown to exhibit sex-specific ageing trajectories (25, 33). Thus, this step allows for a better understanding of variability between the sexes (34). The healthy group was further split into 80% training (F = 6,000, M = 5,346), 10% validation (F = 750, M = 669), and 10% test sets (F = 750, M = 669). For this split, the groups of healthy subjects were stratified by age to ensure representative coverage across the ageing spectrum available in the UK Biobank. The training data from the healthy subjects were then used to train the machine learning models needed to estimate the BAG and HAG in the cardiovascular disease groups (see below). The validation set was utilized in machine model training for early stopping as well as for the age gap bias correction procedure carried out (see section Bias Correction). All data groups remained the same throughout this work for ease of comparability between BAG and HAG.

For further splitting of the subjects with diagnosed cardiovascular diseases (F = 2,304, M = 2,925) “Phecodes” (35), which group International Classification of Diseases Revision 10 (ICD10) codes into interpretable disease classifications, were used. Disease subgroups with fewer than 30 participants were excluded to ensure statistical robustness of the results. This resulted in 36 sex-specific cardiovascular disease test groups (*e.g.,* hypertension, heart valve disorders, congestive heart failure, *etc.*). The full list of disease subgroups, along with their associated Phecodes and ICD-10 codes, is presented in Table 1.

**Table 1:** Demographic information of the subgroups under examination.

Subgroup	Sex	*N*	Mean age
Healthy holdout test	F	728	62.62
	M	630	62.67
Abnormal heart sounds	F	33	63.18
Cardiac conduction disorders	F	71	66.31
	M	179	68.38
Cardiomegaly	M	72	65.83
Carditis	M	33	62.94
Congestive heart failure	F	30	66.93
	M	80	69.15
Elevated blood pressure reading without diagnosis of hypertension	M	44	65.43
Heart valve disorders	F	70	69.49
	M	116	67.76
Hemorrhoids	F	341	62.74
	M	382	63.58
Hypertension	F	1,160	66.78
	M	1,733	66.98
Hypotension	F	109	65.55
	M	86	67.78
Nonspecific chest pain	F	95	65.00
	M	87	63.44
Non-cerebral aneurysms	M	38	67.29
Other disorders of circulatory system	F	190	65.64
	M	241	67.03
Other forms of chronic heart disease	F	30	67.93
	M	84	66.27
Paroxysmal tachycardia, unspecified	F	356	65.51
	M	512	66.12
Phlebitis and thrombophlebitis	M	31	63.00
Pulmonary heart disease	F	52	66.67
	M	93	65.45
Raynaud's syndrome	F	58	64.43
	M	49	66.92
Rheumatic disease of the heart valves	F	42	69.12
	M	54	67.87
Unstable angina intermediate coronary syndrome	F	291	67.24
	M	737	67.81
Varicose veins	F	313	64.51
	M	206	65.17

### 2.2 Brain age prediction

To calculate the brain age gap (BAG), T1-weighted MRI scans (resolutio*n* = 1 × 1 × 1 mm^3^, field of view = 208 × 256 × 256 mm^3^) were used. All T1-weighted MRI scans underwent preprocessing using an in-house developed pipeline (12). This preprocessing included non-local means filtering (36), skull stripping using HDBET (37), N4 bias field correction (38), and affine registration using ANTs (39) to the MNI atlas (40). These steps ensured that the input images were consistent and suitable for the subsequent machine learning analyses. The full T1-weighted images were selected for this purpose, as it has been shown that this is the best and most readily available single imaging modality for brain age prediction (41). Moreover, the whole images were used without any explicit feature extraction to enable a fully data-driven pipeline that overcomes potential biases introduced by the selection of a specific parcellation scheme used for feature extraction.

**Figure 1:**
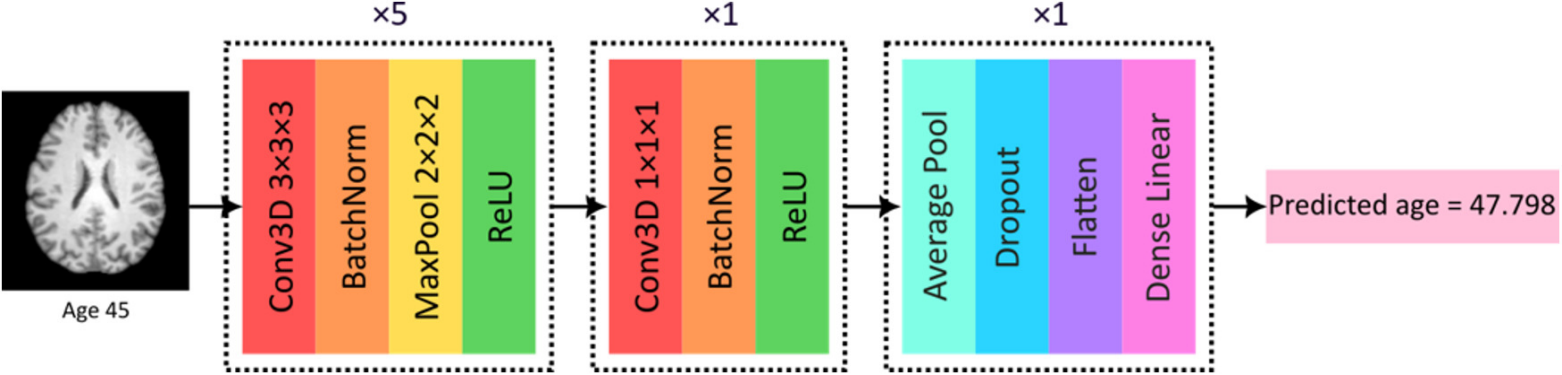
CNN model used in this work for biological brain age prediction using T1-weighted MRI. BatchNorm, batch normalization; Conv3D, 3D convolutional layer; MaxPool, max pooling; ReLU, rectified linear unit. Reproduced with permission from “CNN model used in this work for biological brain age prediction using T1w MRI” by Elizabeth Mcavoy, Emma A. M. Stanley, Anthony J. Winder, Matthias Wilms and Nils D. Forkert, licensed under CC BY-NC-ND.

For biological brain age prediction, a 3D convolutional neural network (CNN) was employed, as illustrated in Figure 1 (12, 14). This deep learning model is based on the widely validated SFCN model (42), which achieved competitive results predicting brain age using UK Biobank data in the past. The CNN model, trained using the data from healthy subjects, outputs a predicted biological brain age for each individual based on the learned patterns in brain structure associated with healthy age-related changes. Two models, one for females and one for males, were trained using the T1-weighted MRIs from the healthy datasets in this work. The trained CNN models were used for prediction and BAG calculation of the holdout test sets of healthy subjects, as well as for the subjects with cardiovascular diseases, whereas the difference between the predicted brain age and the individual's chronological age provides the BAG.

### 2.3 Heart age prediction

The heart age gap (HAG) model used in this work was developed using tabulated structural and functional cardiac features derived from 4D cardiac MRI (31) and pulse wave analysis (PWA) (43). Briefly described, the cardiac MRI features were previously automatically extracted using machine learning algorithms from cine MRI scans in the short-axis, long-axis, and aortic views (31) with manual quality control performed before uploading the data to the UK Biobank repository. These cardiac MRI features included chamber-specific volumes, areas, distensibility, strain, ejection fractions, as well as PWA features, including cardiac index, blood pressures, and cardiac output. The PWA data also included a flag for plausibility of the recorded output as determined by a medical professional, which was used for quality control in this work, wherein only data from subjects with plausible recordings were included. All features used were readily available from the UK biobank as part of “PWA” and “Cardiac and aortic function #1” data categories. Only features directly related to heart function were retained, resulting in a final set of 103 features that can be found in the supplementary material.

**Figure 2:**
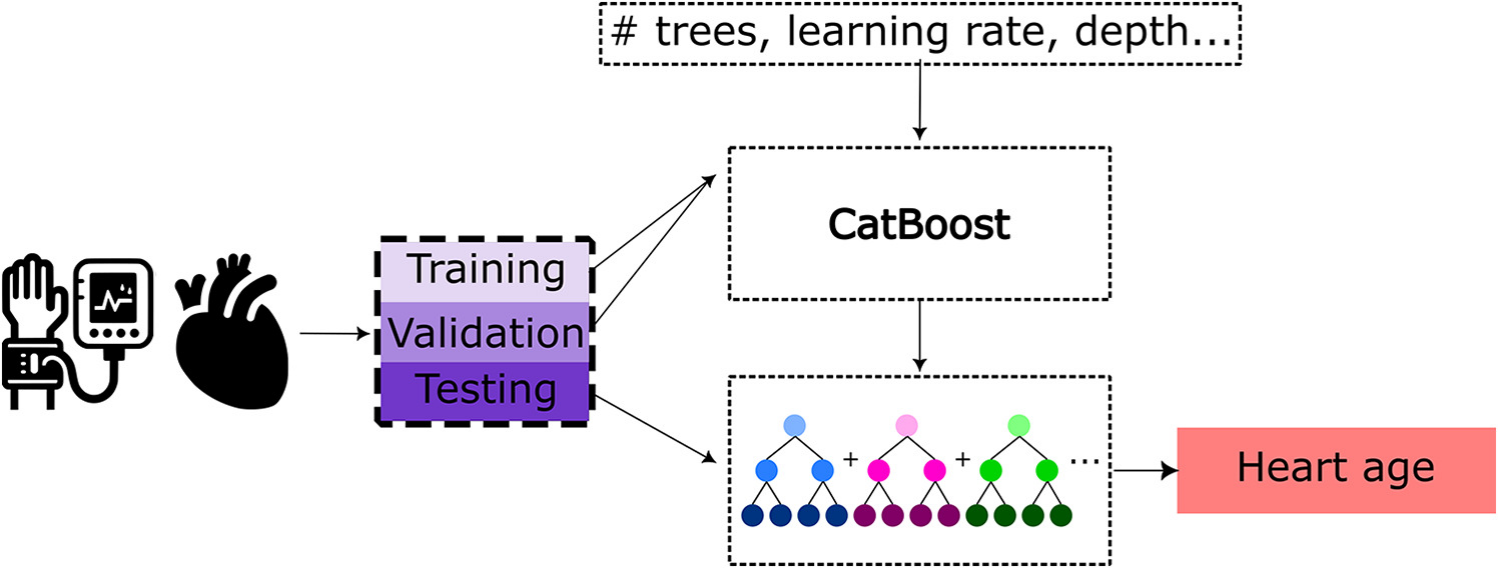
Pipeline for heart age prediction using a CatBoost model based on cardiac MRI-derived features and pulse wave analysis features as the input. Training and hyperparameter tuning of the CatBoost model were performed using Optuna utilizing the training and validation sets, respectively.

To predict the HAG from these tabulated features, a CatBoost tree model (44) with the hyperparameters tuned using Optuna (45) was implemented following the HAG work by Shah et al. (25) The pipeline for this is illustrated in Figure 2. CatBoost is a tree-based gradient boosting algorithm that performs well on tabular data and has shown strong performance in cardiac age prediction using cardiac MRI features and the same hyperparameter tuning framework as used in this work (25). The input groups for training, validation, testing, and cardiac disease subgroup subjects are the same as those used for the BAG prediction model. The model outputs the predicted biological heart age for each individual, wherein the difference between this predicted biological age and chronological age constitutes the HAG. Sex-based differences were observed in some cardiac metrics, and to account for this, separate models for males and females were trained, in the same fashion and using the same patient data splits as done for the brain age prediction task to enable full comparability. The trained CatBoost models were used for biological heart age prediction and HAG calculation of the holdout test sets of healthy subjects, as well as for the subjects with various cardiovascular diseases.

### 2.4 Bias correction

Age gap calculation using machine learning has been shown to typically be incorrectly correlated with chronological age in many cases due to regression-to-the-mean effects, where younger individuals tend to be predicted older and vice versa, independent of the model and data used (46, 47). To correct for this potential bias, a widely used bias correction step (46) was applied. To this end, ordinary least squares linear models were trained using the validation sets of healthy participants and used to “regress out” the influence of chronological age. This correction was independently optimized and applied to both models for HAG and BAG computation, respectively.

### 2.5 Statistical analysis

Statistical comparisons were performed to examine how the age gaps of the cardiovascular disease groups differ from the age gap distributions of the healthy cohort, as well as how BAG and HAG differ within each cardiovascular disease group. In the healthy test set, it is expected that the mean BAG and HAG will be approximately 0 and that any deviations will be randomly distributed. Moreover, it is expected that the cardiovascular disease groups will differ from healthy ageing with respect to the HAG, while significant differences in the BAG suggest that the cardiovascular disease investigated also affects brain ageing. Cardiovascular diseases where the BAG and HAG distributions are not or only weakly correlated and significantly different from each other may suggest that the cardiovascular disease can cause non-uniformity in ageing along the heart-brain-axis. Therefore, Mann–Whitney U-tests were used to compare group means to the healthy holdout test set. To explore how HAG and BAG aligned within individuals, paired Wilcoxon signed-rank tests were conducted for each of the cardiovascular disease groups. Additionally, to investigate if the directions of potential BAG and HAG differences in the disease groups were correlated, Spearman correlation coefficients were calculated, with values less than 0.3 indicating poor, 0.3–0.5 fair, 0.6–0.8 moderately strong, and above 0.8 very strong correlations (48). All statistical tests were bootstrapped with 10,000 samples to improve reliability and to account for variability. The mean *p*-values from these resampling tests were reported. In the determination of significance while correcting for multiple comparisons, Benjamini-Hochberg (49) corrections for false discovery were applied, with a *p*-value and q-value of 0.05.

## 3 Results

**Figure 3:**
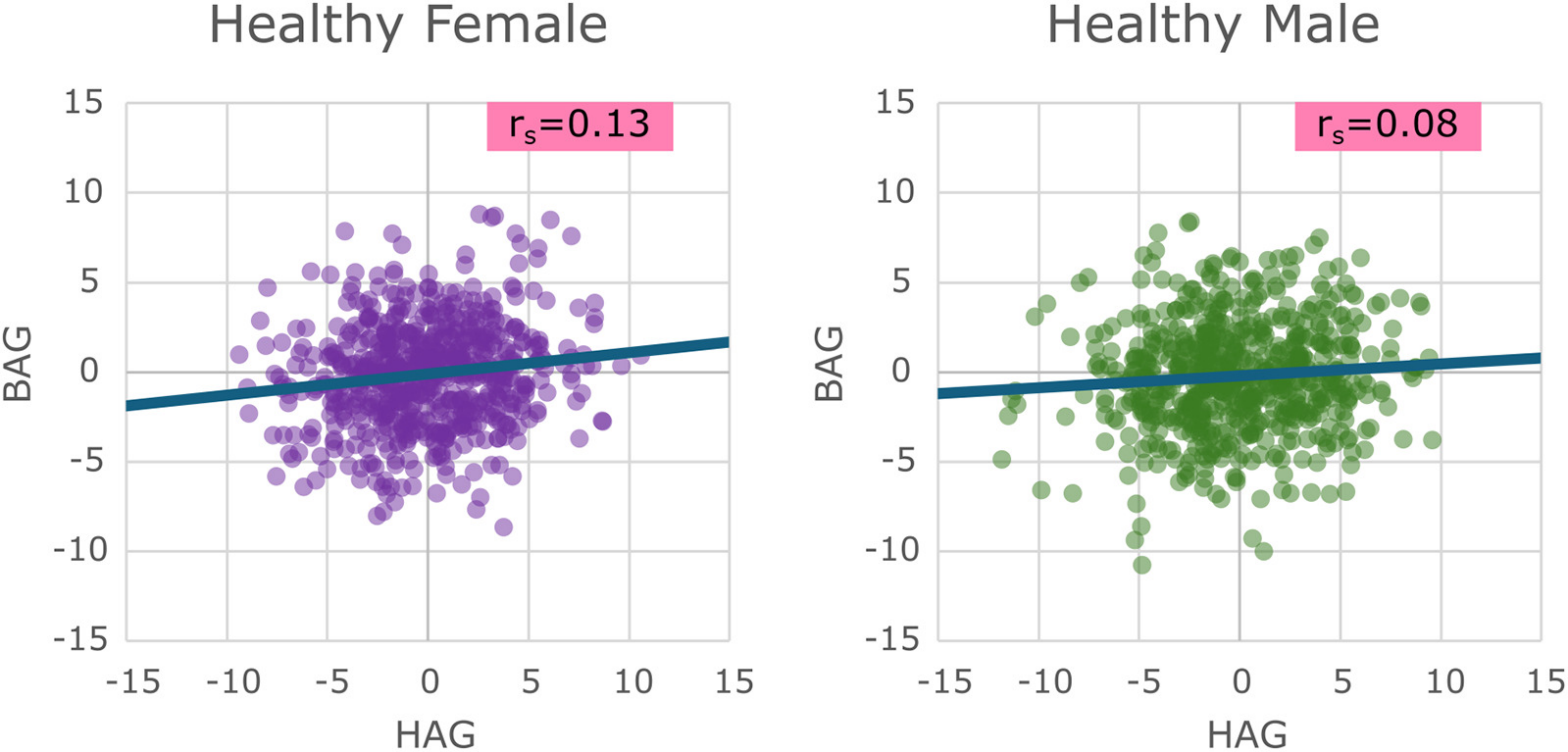
Scatter plots of the healthy test sets for males and females showing their brain age gap vs. their heart age gap, with a line of best fit showing the overall trend present.

For the sex-specific healthy cohorts before bias correction, both BAG and HAG showed moderately strong correlations (r) with chronological age [BAG: Female (F) r = −0.525, Male (M) r = −0.597, HAG: F r = −0.693 M r = −0.692], which diminished after bias correction (BAG: F r = 0.052, M r = 0.079, HAG: F r = 0.001, M r = 0.058), as expected. Thus, this correction ensured that the measures reflect biological ageing rather than simply chronological age. Scatter plots of the BAG vs. HAG for the healthy test groups can be seen in Figure 3.

Spearman correlation between BAG and HAG for the healthy test sets was very poor (F: r = 0.13, M: r = 0.08). The resulting mean age gaps and mean absolute errors (MAE) are provided in Table 2. It can be seen that the mean age gaps for males and females in both HAG and BAG are (as expected) approximately 0.

**Table 2:** Age gaps for healthy test sets showing the means and mean absolute error (MAE).

Healthy sets	Female		Male	
	Mean	MAE	Mean	MAE
HAG	−0.048	2.747	−0.159	3.035
BAG	−0.114	2.266	−0.218	2.404

Next, the following comparisons were conducted for each of the 36 disease groups:

(1) BAG for healthy subjects (BAG_Healthy_) vs. BAG values for each disease group (BAG_Disease_)
(2) HAG for healthy subjects (HAG_Healthy_) vs. HAG values for each disease group (HAG_Disease_)
(3) BAG values for each disease group (BAG_Disease_) vs. HAG values for the same disease group (HAG_Disease_)

**Figure 4:**
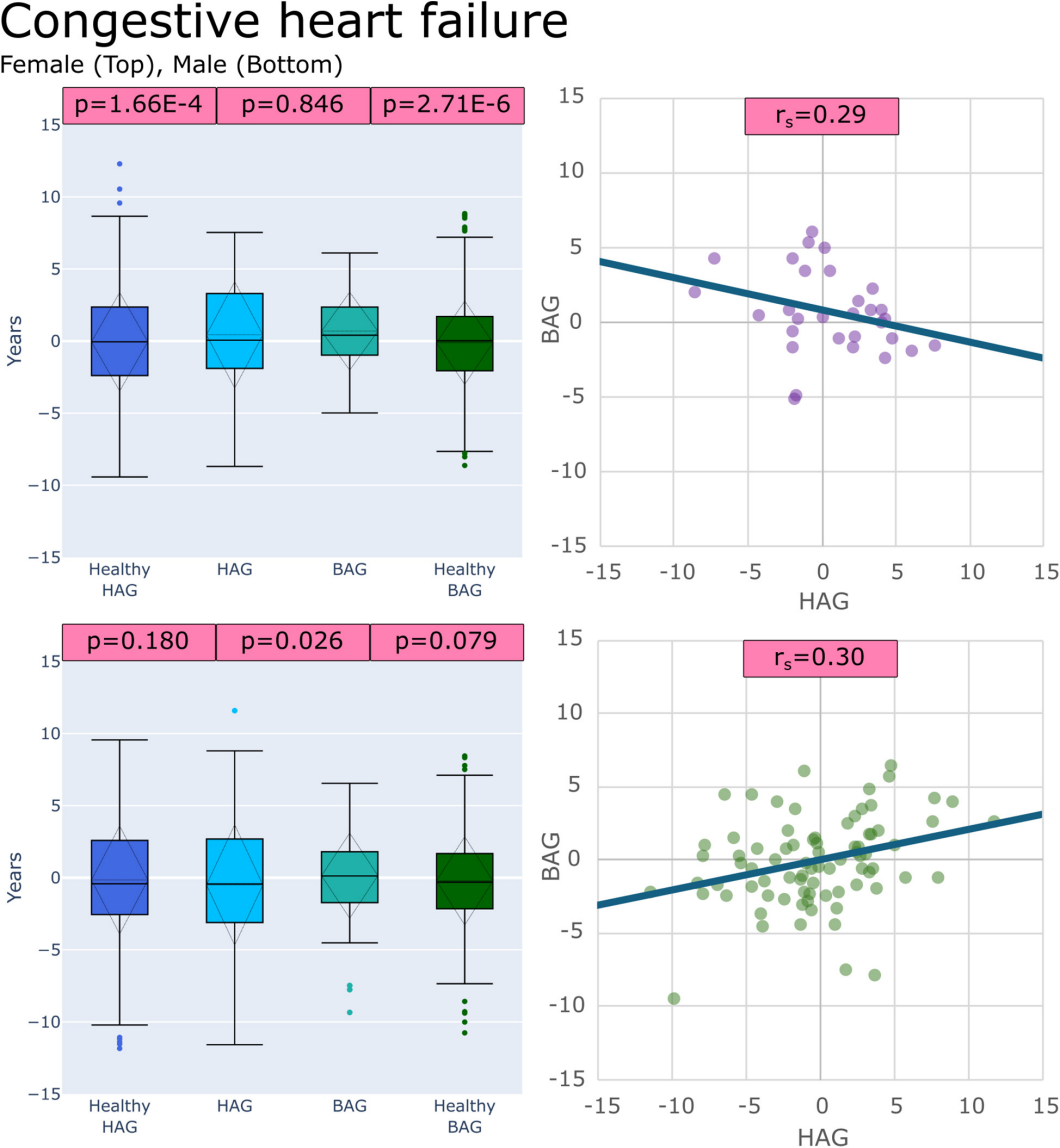
Plots of age gaps for patients with congestive heart failure, female (top), male (bottom), (left) box plots comparing distributions to healthy test groups with *p* values from tests (left to right): (HAG_Healthy_ vs. HAG_Disease_), (BAG_Disease_ vs. HAG_Disease_) and, (BAG_Healthy_ vs. BAG_Disease_) (right) scatter plot of the patient-specific BAG and HAG with a line of best fit.

**Figure 5:**
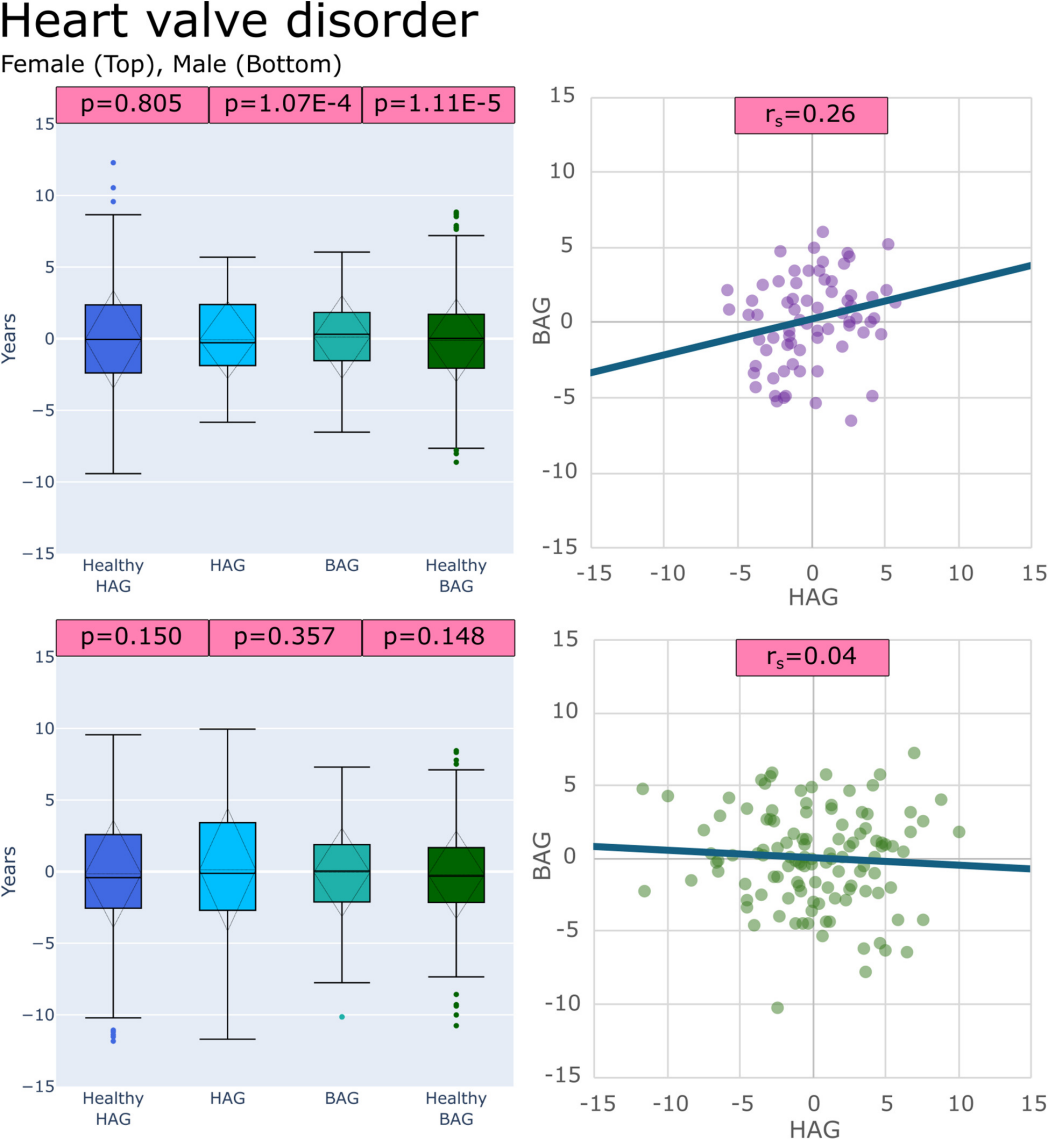
Plots of age gaps for patients with heart valve disorder, female (top), male (bottom), (left) box plots comparing distributions to healthy test groups with *p* values from tests (left to right): (HAG_Healthy_ vs. HAG_Disease_), (BAG_Disease_ vs. HAG_Disease_) and, (BAG_Healthy_ vs. BAG_Disease_) (right) scatter plot of the patient-specific BAG and HAG with a line of best fit.

These comparisons of BAG and HAG distributions revealed some significant differences. Specifically, out of the 36 sex-specific disease groups under investigation, 24/36 showed a significant difference in at least one of the three test categories (BAG_Healthy_ vs. BAG_Disease)_, (HAG_Healthy_ vs. HAG_Disease_), or (BAG_Disease_ vs. HAG_Disease_) after correction for multiple testing. Box plots showing the results of these comparisons for congestive heart failure and heart valve disorder groups are provided in Figures 4,5, respectively. Box plots for all other disease groups can be found in the Supplementary Material.

21/36 sex-specific disease groups showed significantly different (BAG_Healthy_ vs. BAG_Disease_) distributions, 13/36 showed significantly different (HAG_Healthy_ vs. HAG_Disease_) distributions, and 11/36 showed that (BAG_Disease_ vs. HAG_Disease_) distributions were significantly different. Table 3 shows all disease groups examined with detailed results of the comparison tests, (BAG_Healthy_ vs. BAG_Disease_), (HAG_Healthy_ vs. HAG_Disease_), and (BAG_Disease_ vs. HAG_Disease_). The only sex-specific disease groups that showed no differences in all comparisons were for females with hemorrhoids, unstable angina intermediate coronary syndrome, and varicose veins and for males with cardiomegaly, congestive heart failure, heart valve disorders, hemorrhoids, hypotension, other forms of chronic heart disease, nonspecific chest pain, and rheumatic disease of the heart valves.

**Table 3:** Cardiovascular diseases group comparisons significant results in (BAG_Healthy_ vs. BAG_Disease_), (HAG_Healthy_ vs. HAG_Disease_), and (BAG_Disease_ vs. HAG_Disease_), Spearman correlation coefficient BAG and HAG.

Disease group	Sex	BAG_Healthy_ vs. BAG_Disease_	HAG_Healthy_ vs. HAG_Disease_	BAG_Disease_ vs. HAG_Disease_	Absolute Spearman Correlation
Abnormal heart sounds	F	**0.015**	0.575	0.240	0.15
Cardiac conduction disorders	F	**0.002**	**1.35E-07**	0.114	0.08
	M	**6.16E-05**	**0.008**	**8.17E-11**	0.06
Cardiomegaly	M	0.557	0.547	0.908	0.16
Carditis	M	**3.69E-04**	0.824	0.029	0.13
Congestive heart failure	F	**2.71E-06**	**1.66E-04**	0.846	0.29
	M	0.079	0.180	0.026	**0.30**
Elevated blood pressure reading without diagnosis of hypertension	M	**0.003**	**2.50E-04**	0.598	0.08
Heart valve disorders	F	**1.11E-05**	0.805	**1.07E-04**	0.26
	M	0.148	0.150	0.357	0.04
Hemorrhoids	F	0.229	0.840	0.225	0.09
	M	0.043	0.932	0.078	0.09
Hypertension	F	**4.90E-06**	**6.16E-07**	0.572	0.10
	M	**1.12E-09**	**5.79E-05**	0.638	0.04
Hypotension	F	0.039	**1.84E-06**	**6.10E-13**	0.12
	M	0.063	0.503	0.774	0.06
Non-cerebral aneurysms	M	**5.28E-08**	**5.52E-06**	0.577	0.14
Nonspecific chest pain	F	**0.003**	**4.79E-04**	0.128	0.07
	M	0.472	0.232	0.088	**0.34**
Other disorders of circulatory system	F	0.933	**0.004**	**0.002**	0.08
	M	**5.65E-05**	0.682	**0.006**	0.02
Other forms of chronic heart disease	F	0.597	**1.87E-05**	**5.45E-10**	0.10
	M	0.053	0.585	0.034	0.07
Paroxysmal tachycardia, unspecified	F	0.530	**0.016**	0.162	0.07
	M	**0.016**	0.070	0.629	0.04
Phlebitis and thrombophlebitis	M	**1.37E-13**	0.280	**0.010**	**0.33**
Pulmonary heart disease	F	**0.002**	0.442	0.434	0.05
	M	**1.27E-06**	0.163	**0.003**	0.07
Raynaud's syndrome	F	**0.021**	0.184	0.553	0.01
	M	**0.008**	0.496	**0.006**	0.01
Rheumatic disease of the heart valves	F	**1.13E-04**	0.550	**0.007**	0.02
	M	0.928	0.240	0.168	0.09
Unstable angina intermediate coronary syndrome	F	0.815	0.711	0.931	0.07
	M	**3.08E-04**	0.026	0.566	0.09
Varicose veins	F	0.479	0.105	0.176	0.12
	M	**0.004**	**0.012**	**1.06E-07**	0.17

Bold indicates significant results after multiple testing correction.

Spearman correlation was used to evaluate the relationship between BAG and HAG, exploring whether differences are correlated in a more general way than the (BAG_Disease_ vs. HAG_Disease_) test. In this analysis, only 3/36 groups showed marginally fair (|0.3–0.5|) correlations between BAG and HAG, with the majority of cardiovascular diseases showing only poor correlations (<|0.3|).

## 4 Discussion

Overall, the results of this study show that the heart age gap and the brain age gap are two distinct biomarkers that do not appear to be correlated in the presence of cardiovascular diseases. Instead, in combination, they provide insight into how the heart and brain age differently in the presence of different cardiovascular diseases.

The heart and brain age prediction models implemented in this work performed on par or better than the results described in the original contributions presenting the model architectures used (25, 50). More precisely, the models developed in this work led to an improvement in the previously described mean absolute error (MAE) from 4.21 years (25) compared to an MAE = 2.75 years for females and MAE = 3.04 years for males for the heart age prediction models in our work, and a previously described MAE = 2.47 years (50) compared to an MAE = 2.27 years for females and MAE = 2.40 years for males for the brain age prediction models in our work. Thus, the models performed slightly differently between the sexes. In both cases, the mean absolute error was lower for the biological age prediction in the female cohort. This presents an opportunity for further investigation into any additional confounding variables that may explain the sex-specific differences found for the healthy brain age prediction as well as the heart age prediction.

Previous studies investigating healthy subjects found correlations between heart and brain age gaps (9, 51). However, when investigating patients with diseases, it gets more complicated. For example, it has been reported that there is a divergence of heart and brain ages in patients with chronic diseases (9), while an investigation into the underlying genetic architecture of heart and brain ages also revealed a very weak correlation between the heart and brain ages (10). Similarly to those results from the literature, our work shows a divergence between the heart and brain age gaps in the presence of the specific cardiovascular diseases investigated in this work. This finding suggests that a heterogeneity in the ageing biomarkers and the trajectories they measure exists, and that the heart and brain are not always ageing in sync in the presence of cardiovascular diseases. Thus, the combination of these two biomarkers allows for a quantitative analysis of how a cardiovascular disease affects both the heart and brain in individual patients. The fact that 24 out of 36 disease groups showed significant differences in at least one of the comparisons of BAG and HAG emphasizes the value of this dual biomarker approach. Age gaps can be used for the analysis of the interplay and patterns between two or more organs of the human body, whereas the combination of BAG and HAG allows for a better understanding of how different cardiovascular diseases affect the heart and the brain from a biological ageing perspective.

Another interesting finding of this study is that for the sex-specific groups, multiple cardiovascular diseases exhibited differences between the female and male cohorts. Of the 16 female disease groups, 56% showed a BAG distribution that was different from the healthy group, 50% a HAG distribution that differed from the healthy group, and 31% showed significant differences between the BAG and HAG distributions. In contrast to that, for the 20 cardiovascular disease male groups investigated, 60% showed a BAG distribution that differs from the healthy test cohort, 25% showed a HAG distribution different from the healthy group, and 30% showed significantly different HAG and BAG distributions. This observation suggests that, depending on the disease and sex status, cardiovascular diseases manifest differently in the imaging features related to brain and heart ageing. This finding aligns with previous research showing that men and women experience healthy cardiovascular and neurological ageing differently (25, 27, 33, 52). For example, in healthy cardiovascular ageing, a feature that differs greatly depending on sex is the normalized left ventricular mass and function (53). In brain ageing, there are similar trends, with sex and menopause, for example, being associated with changes in grey matter, white matter, and ventricular volumes (33). Another example of sex-related differences in the ageing process of the brain and heart is the presence of cardiac plaques. In those patients, women have been shown to exhibit brain atrophy equivalent to 8.8 years while men exhibit white matter hyperintensities equivalent to 6.5 years (54). These sex-specific differences in the ageing biomarkers warrant further causality-based investigations (55), which may help to explore if there are certain biological and sex-specific protective features that may be useful in the future. This disease- and sex-specific interaction highlights the importance of personalized medicine and investigation into how diseases manifest in different sexes.

Previous studies showed an increase in parenchymal brain matter loss or atrophy in patients with increased cardiovascular stress blood biomarkers (56). Different heart diseases have also been associated with structural brain changes, with a majority of patients with such diseases exhibiting differences in brain atrophy and white matter hyper- and hypo-intensities compared to healthy participants (57, 58). While brain atrophy and white matter hyper- and hypo-intensities can be observed in normal brain ageing, they are also associated with dementia if occurring earlier in life (33, 59–62). Furthermore, it has been reported that subjects with hypertension (63) and abnormalities in blood pressure, even without clinical hypertension, exhibit increased brain atrophy and are at increased risk for dementia (64). These previous findings are in line with the results of this work, where subjects with hypertension (M, F) and elevated blood pressure (M) exhibit both BAG and HAG differences when compared to the healthy cohort. Another result in the present study that is in line with previous research is that females with heart valve disorders exhibit a difference in the BAG distribution when compared to healthy subjects. Within this context, it has been previously reported that 87% of patients with heart valve disorders exhibit increased white matter hyperintensities, a sign often associated with accelerated brain ageing (58). With respect to the association between cardiovascular diseases and the brain age gap, previous work has found that, in particular, the ventricular regions of the brain have high importance and are used by deep learning models for the brain age prediction task (32). This previous work also found that the regions utilized by a deep learning model for brain age prediction did not significantly differ in patients with cardiovascular diseases compared to healthy participants, which suggests that deep learning models trained for brain age prediction primarily analyze and make use of normal brain aging patterns (32). Previous work related to the HAG has found that the features derived from cardiac MRI, specifically aortic and left heart chamber features, have high importance for the heart age prediction task when trained on data from healthy subjects (25). Further analysis into specific feature-wise correlation between the brain and heart age in future studies would enable a more in-depth biological interpretation of the heart and brain ages in a wide range of cardiovascular diseases.

However, despite the fact that some of the results found in this work are generally in line with previous disease-specific findings, the low correlation of the BAG and HAG in patients with cardiovascular diseases, although previously reported for other disease types (9, 10), may seem surprising given an assumed heart-brain axis. The fact that the HAG and BAG are only weakly or negligibly correlated in the cardiovascular disease groups examined raises questions about the generalizability of the heart-brain axis in reference to age gap biomarkers. Many theories to explain how the heart and the brain are anatomically and functionally linked have been proposed in the past, such as the neuro-endocrine-heart axis and neuro-immune-heart axis hypotheses (65–67). Particularly, the neuro-immune-heart axis theory could be a possible explanation for a link between the three diseases which showed a higher correlation between the BAG and HAG. More precisely, phlebitis refers to an inflammation of the veins (68), while chronic heart failure has been associated with increased inflammatory biomarkers (69), and non-specific chest pain is a possible symptom related to inflammation of the chest wall. Further investigation into the causality of the BAG and HAG in the future could help to better explore the relationship between the ageing biomarkers and cardiovascular diseases. Following this, it may be expected that the heart-brain axis manifests differently in men and women, likely because hormonal and immune responses differ between the sexes (70). Due to the nature of the age distribution within the UK Biobank (46–82 years), a majority of the female subjects are possibly menopausal or post-menopausal. Thus, further investigation of the hormonal balance present in men and women, as well as possible investigation of inflammatory markers, could allow for more specific future analyses of correlations and causation of the BAG and HAG differences in women and men. Both immune and endocrine responses can vary drastically in duration and severity of the response. This could be an explanation for why only low correlations between the HAG and BAG distributions were found in this study. Thus, further work using longitudinal data is needed to explore how these biomarkers change over time for individuals with cardiovascular disease to investigate if the age gaps observed become in sync over time.

Overall, our results suggest that the combination of BAG and HAG as biomarkers provides a more comprehensive view of biological ageing compared to using either metric alone. The BAG captures neuroanatomical changes, while the HAG reflects cardiovascular function and vascular health. This has the potential to explain why some cardiovascular diseases can cause neurological problems and vice versa. Together, these biomarkers clinically offer the possibility of a holistic assessment of cardiovascular disease, making them valuable tools for identifying individuals at high risk for both cardiac and neurological decline without the assumption that the two systems will always be in sync.

### 4.1 Limitations

This study has several limitations that should be considered when interpreting the findings. First, the reliance on data from the UK Biobank, while providing a well-organized dataset of deeply phenotyped subjects, may limit the generalizability of results to other populations due to potential demographic and health-related biases within the underlying study cohort. Thus, applying and evaluating the model's performance in more diverse cohorts that also cover a larger age range than the participants included in the UK Biobank would increase its future generalizability to different demographics in clinical practice. Additionally, exploration of different representative measures/variables of heart and brain health, such as those obtained from low-field MRIs, or electrocardiograms and electroencephalograms, that are more easily obtained clinically than MRI, would be beneficial in the future to enable affordable and continuous monitoring of individuals in large populations.

This study specifically utilized physiological measurements and imaging for the prediction of heart and brain ages. Thus, further investigation into additional (confounding) variables that could explain higher or lower age gaps would enable a better understanding of how these age gaps present clinically in patients. Particularly, variables such as socioeconomic status, medications, lifestyles, and other disease-related factors, should be investigated in the future to better understand their potential influence on the results.

Sex-specific models were employed to minimize the influence of sex as a confounding variable. The findings of this study suggest that the sexes differ in HAG and BAG in cardiovascular disease. Since healthy ageing differs between sexes and cardiovascular diseases manifest differently between sexes, further research is needed to investigate more specific features and how they differ between sexes. This research would illuminate the nuanced roles that sex may play in the biological ageing of the heart and brain in the presence of cardiovascular diseases, potentially uncovering distinct ageing trajectories and risk factors unique to each sex.

Finally, the study design only captures deviations in brain and heart ageing at a single time point, as well as defining the disease information as present or absent at that time point. Given the possibility of a delay in the response of the heart-brain axis through the immune or endocrine systems (65–67), duration of disease should be explored as an additional variable of interest in the future. Longitudinal studies would provide a more comprehensive understanding of individual ageing trajectories, which could help to identify deviations that could indicate early disease onset or accelerated ageing over time. Such an approach could reveal early biomarkers for at-risk individuals and provide insights into the dynamics of heart-brain ageing as diseases progress.

## 5 Conclusion

This work shows the need for a more holistic approach with respect to the evaluation of cardiovascular diseases. The lack of generalizability of the heart-brain axis using the age gap biomarkers suggests that monitoring both brain and heart health is essential for understanding the full impact of cardiovascular disease on individual patients. Significant differences observed across 24 out of 36 sex-specific cardiovascular disease groups show that these diseases may affect both organs but at different rates, as none of the BAG and HAG were highly correlated with each disease group. Future research could explore whether interventions or behavioral changes targeting one organ system can positively affect the other, offering new avenues for treatment. Additionally, the use of these biomarkers in longitudinal studies could help track disease progression and treatment response over time. Overall, this work highlights the complex and interconnected nature of the heart and brain in subjects with cardiovascular diseases.

## Data Availability

The data analyzed in this study is subject to the following licenses/restrictions: apply to get access to the data through the UK Biobank. Requests to access these datasets should be directed to https://www.ukbiobank.ac.uk/enable-your-research/apply-for-access.
