# Supplementary material for: Biological heart and brain ageing in subjects with cardiovascular diseases
Source: Front Cardiovasc Med. 2025 Jul 7;12:1569423. doi: 10.3389/fcvm.2025.1569423 (PMC12278062; doi:10.3389/fcvm.2025.1569423)
Supplement: Supplementary file 1 [file Datasheet1.pdf]

Supplementary Material

1. List of tabular cardiac features used for heart age prediction

24120	Ascending aorta distensibility
24118	Ascending aorta maximum area
24119	Ascending aorta minimum area
12681	Augmentation index for PWA
22426	Average heart rate
22425	Cardiac index
12702	Cardiac index during PWA
22424	Cardiac output
12682	Cardiac output during PWA
12680	Central augmentation pressure during PWA
12678	Central pulse pressure during PWA
12677	Central systolic blood pressure during PWA
24123	Descending aorta distensibility
24121	Descending aorta maximum area
24122	Descending aorta minimum area
12698	Diastolic brachial blood pressure
12675	Diastolic brachial blood pressure during PWA
12683	End systolic pressure during PWA
12684	End systolic pressure index during PWA
12673	Heart rate during PWA
24113	LA ejection fraction
24110	LA maximum volume
24111	LA minimum volume
24112	LA stroke volume
24104	LV cardiac output
24141	LV circumferential strain AHA 1
24150	LV circumferential strain AHA 10
24151	LV circumferential strain AHA 11
24152	LV circumferential strain AHA 12
24153	LV circumferential strain AHA 13
24154	LV circumferential strain AHA 14
24155	LV circumferential strain AHA 15
24156	LV circumferential strain AHA 16
24142	LV circumferential strain AHA 2
24143	LV circumferential strain AHA 3
24144	LV circumferential strain AHA 4
24145	LV circumferential strain AHA 5
24146	LV circumferential strain AHA 6
24147	LV circumferential strain AHA 7
24148	LV circumferential strain AHA 8
24149	LV circumferential strain AHA 9

24157	LV circumferential strain global
24103	LV ejection fraction
24100	LV end diastolic volume
24101	LV end systolic volume
24181	LV longitudinal strain global
24175	LV longitudinal strain Segment 1
24176	LV longitudinal strain Segment 2
24177	LV longitudinal strain Segment 3
24178	LV longitudinal strain Segment 4
24179	LV longitudinal strain Segment 5
24180	LV longitudinal strain Segment 6
24124	LV mean myocardial wall thickness AHA 1
24133	LV mean myocardial wall thickness AHA 10
24134	LV mean myocardial wall thickness AHA 11
24135	LV mean myocardial wall thickness AHA 12
24136	LV mean myocardial wall thickness AHA 13
24137	LV mean myocardial wall thickness AHA 14
24138	LV mean myocardial wall thickness AHA 15
24139	LV mean myocardial wall thickness AHA 16
24125	LV mean myocardial wall thickness AHA 2
24126	LV mean myocardial wall thickness AHA 3
24127	LV mean myocardial wall thickness AHA 4
24128	LV mean myocardial wall thickness AHA 5
24129	LV mean myocardial wall thickness AHA 6
24130	LV mean myocardial wall thickness AHA 7
24131	LV mean myocardial wall thickness AHA 8
24132	LV mean myocardial wall thickness AHA 9
24140	LV mean myocardial wall thickness global
24105	LV myocardial mass
24158	LV radial strain AHA 1
24167	LV radial strain AHA 10
24168	LV radial strain AHA 11
24169	LV radial strain AHA 12
24170	LV radial strain AHA 13
24171	LV radial strain AHA 14
24172	LV radial strain AHA 15
24173	LV radial strain AHA 16
24159	LV radial strain AHA 2
24160	LV radial strain AHA 3
24161	LV radial strain AHA 4
24162	LV radial strain AHA 5
24163	LV radial strain AHA 6
24164	LV radial strain AHA 7
24165	LV radial strain AHA 8

24166	LV radial strain AHA 9
24174	LV radial strain global
24102	LV stroke volume
12687	Mean arterial pressure during PWA
12679	Number of beats in waveform average for PWA
12676	Peripheral pulse pressure during PWA
24117	RA ejection fraction
24114	RA maximum volume
24115	RA minimum volume
24116	RA stroke volume
24109	RV ejection fraction
24106	RV end diastolic volume
24107	RV end systolic volume
24108	RV stroke volume
12686	Stroke volume during PWA
12697	Systolic brachial blood pressure
12674	Systolic brachial blood pressure during PWA
12685	Total peripheral resistance during PWA

2. Results for all diseases under test and associated Phecodes and ICD10 codes

2.1. Abnormal heart sounds

<table>
<tr><td>Phecode</td><td colspan="3">ICD10 codes</td></tr>
<tr><td>396</td><td colspan="3">R00, R01, R01.0, R01.1, R01.2</td></tr>
<tr><td colspan="4">Female</td></tr>
<tr><td>BAG mean= -0.361</td><td>BAG MAE= 2.126</td><td>HAG mean= -0.160</td><td>HAG MAE= 2.890</td></tr>
<tr><td colspan="2">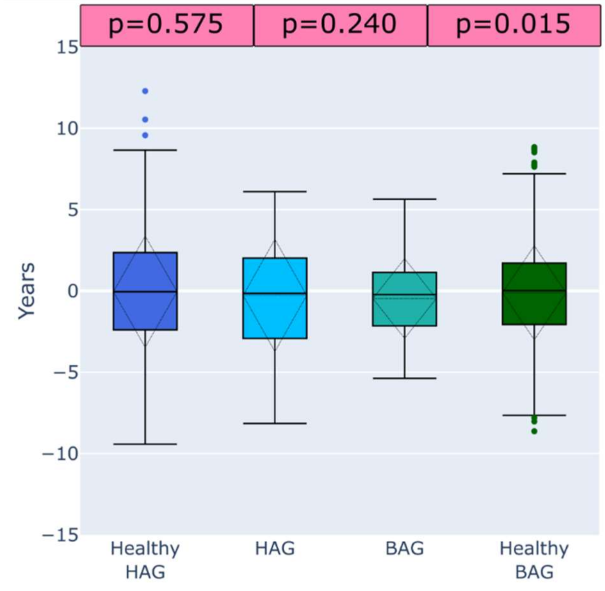
</td><td colspan="2">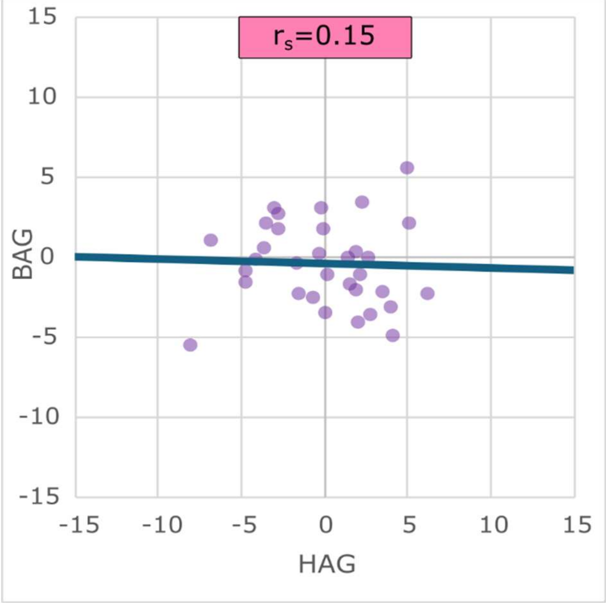
</td></tr>
</table>

2.2. Cardiac conduction disorders

<table>
<tr><td>Phecode</td><td colspan="3">ICD10 codes</td></tr>
<tr><td>426</td><td colspan="3">I44.0, I44.1, I44.2, I44.3, I44.4, I44.5, I44.6, I44.7, I45.0, I45.1, I44.6, I45.2, I45.3, I45.4, I45.5, I45.6, I45.8, I45.9, Z95.0, Z95.9</td></tr>
<tr><td colspan="4" align="center">Female</td></tr>
<tr><td>BAG mean= -0.534</td><td>BAG MAE= 2.321</td><td>HAG mean= -0.988</td><td>HAG MAE= 3.005</td></tr>
<tr><td colspan="2">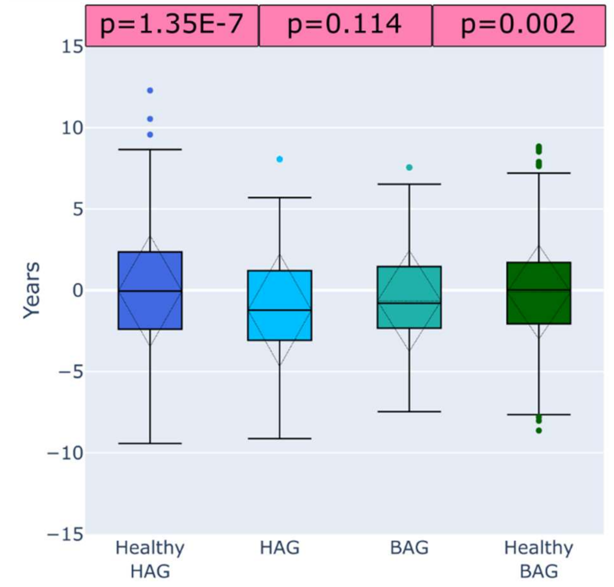
</td><td colspan="2">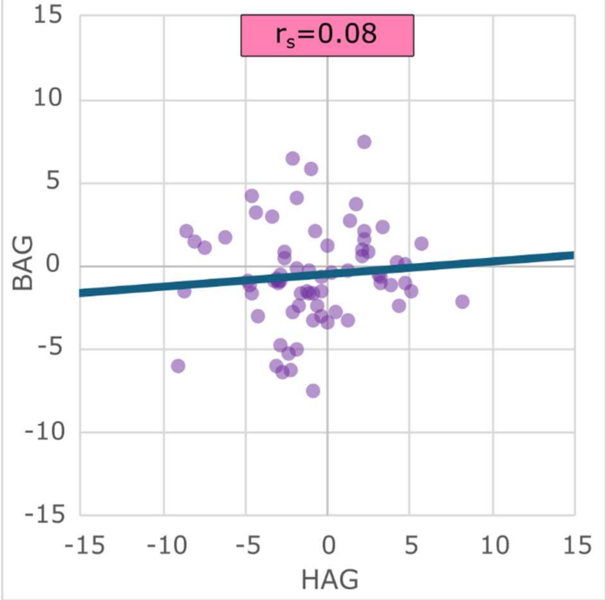
</td></tr>
<tr><td colspan="4" align="center">Male</td></tr>
<tr><td>BAG mean= 0.465</td><td>BAG MAE= 2.322</td><td>HAG mean= -0.452</td><td>HAG MAE= 2.936</td></tr>
<tr><td colspan="2">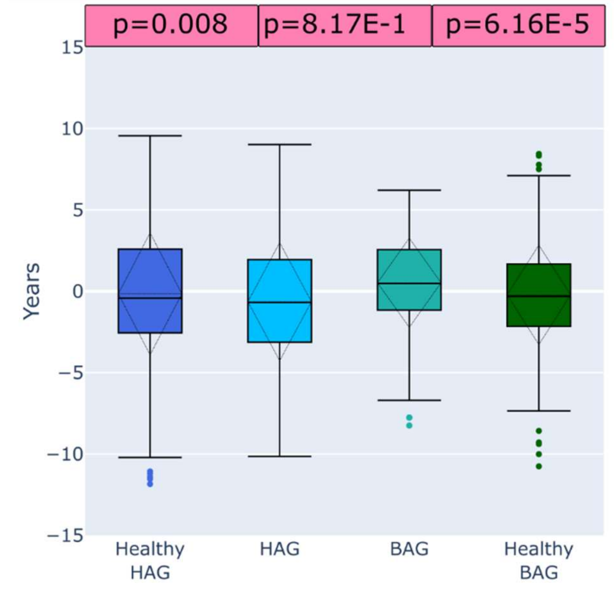
</td><td colspan="2">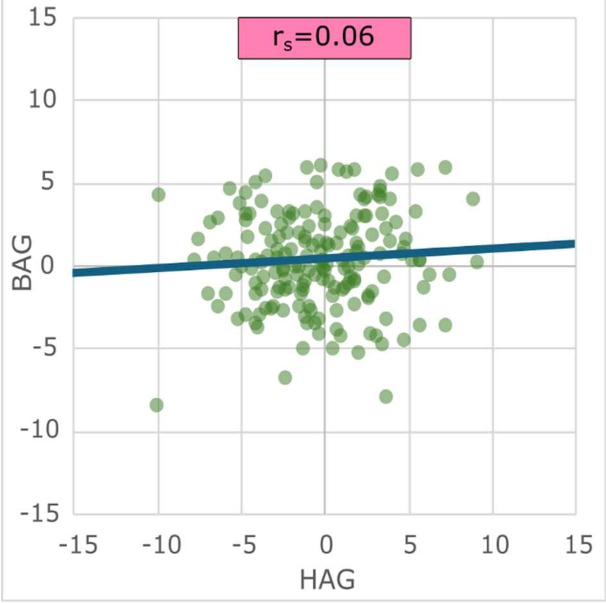
</td></tr>
</table>

2.3. Cardiomegaly

Phecode	***ICD10 codes***		
416	I51.7		
Male			
BAG mean= -0.136	BAG MAE= 2.509	HAG mean= -0.195	HAG MAE= 3.239

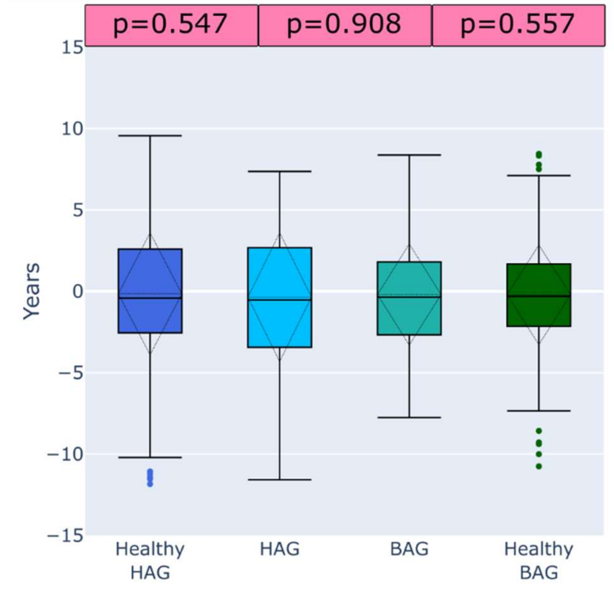

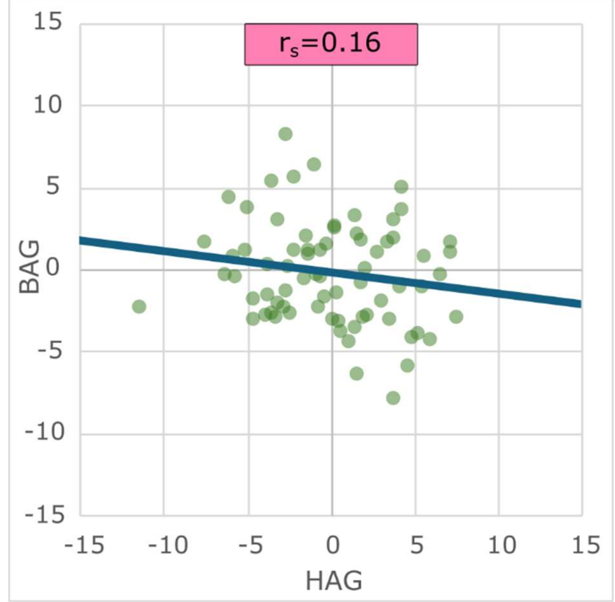

2.4. Carditis

Phecode	***ICD10 codes***
420	A39.5, B33.2, B37.6, I01.0, I01.1, I01.2, I08.8, I08.9, I09.0, I09.2, I30, I30.0, I30.1, I30.8, I30.9, I31, I31.0, I31.1, I31.2, I31.3, I31.8, I31.9, I32, I32.0, I32.1, I32.8, I33, I33.0, I33.9, I38, I40, I40.0, I40.1, I40.8, I40.9, I41, I41.2, I41.8, I51.4

Male

BAG mean= 0.317	BAG MAE= 2.776	HAG mean= -0.409	HAG MAE= 3.892

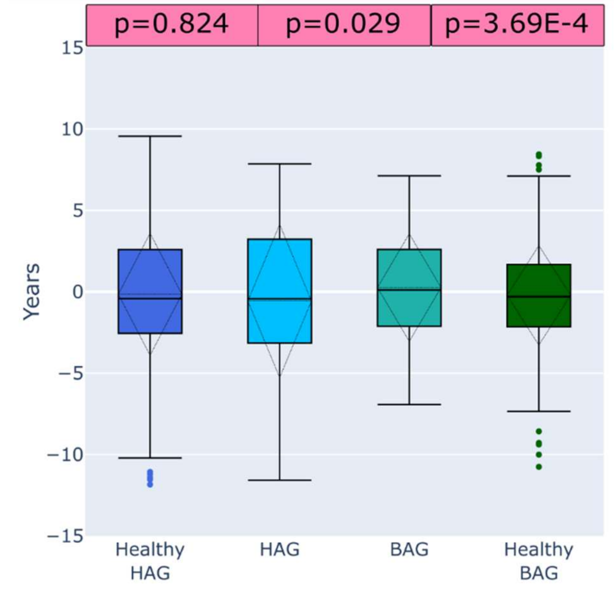

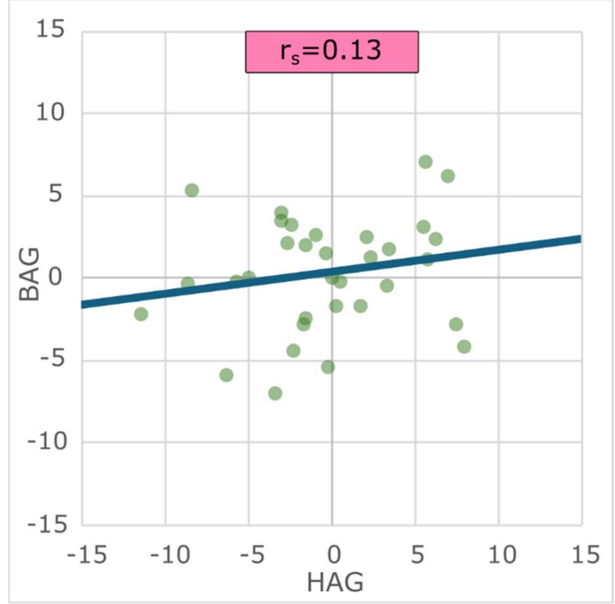

2.5. Congestive heart failure

Phecode	***ICD10 codes***		
428	I50, I50.0, I50.1, I50.9		
Female			
BAG mean= 0.726	BAG MAE= 2.186	HAG mean= 0.474	HAG MAE= 2.939

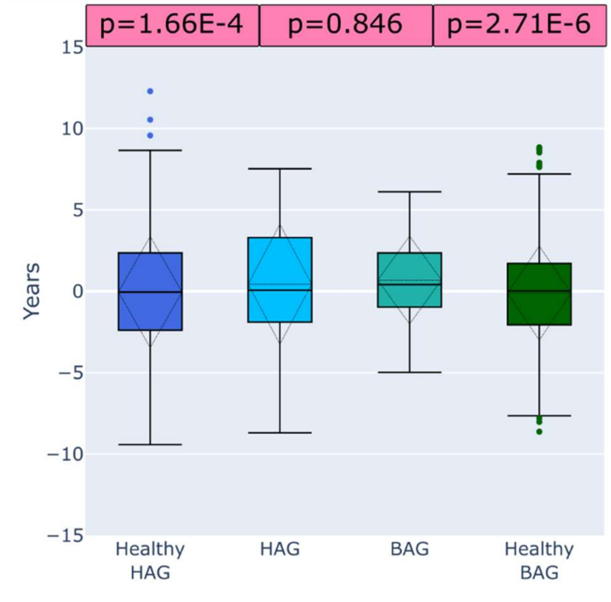

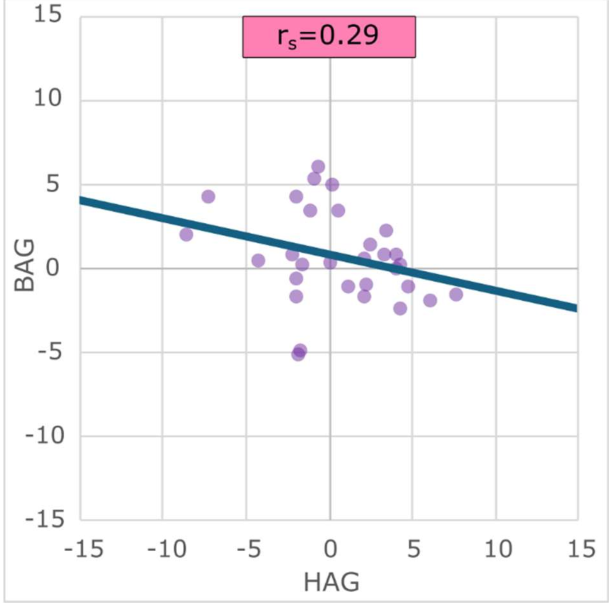

Male			
BAG mean= -0.063	BAG MAE= 2.314	HAG mean= -0.443	HAG MAE= 3.549

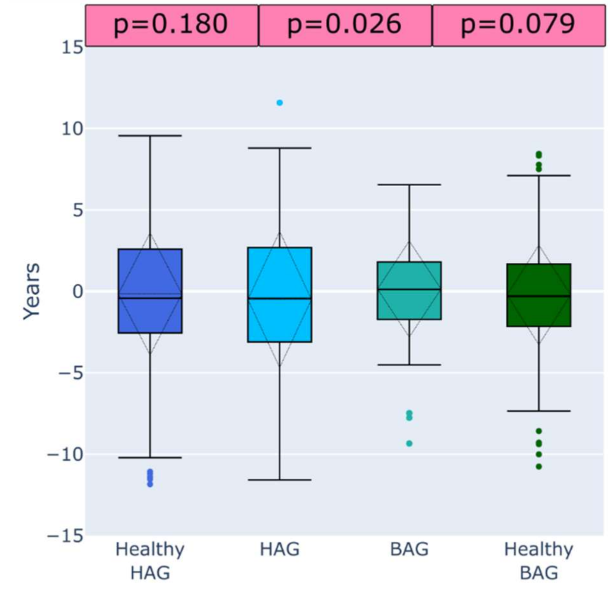

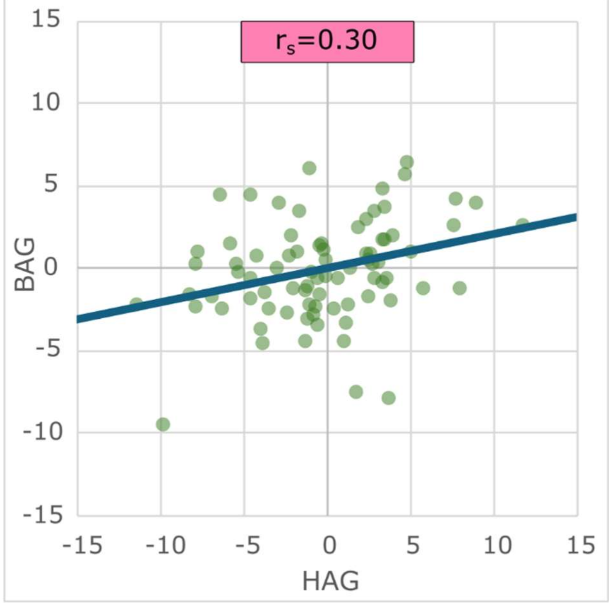

2.6. Elevated blood pressure reading without diagnosis of hypertension

Phecode	***ICD10 codes***		
402	R03.0		
Male			
BAG mean= 0.492	BAG MAE= 2.684	HAG mean= 0.525	HAG MAE= 2.723

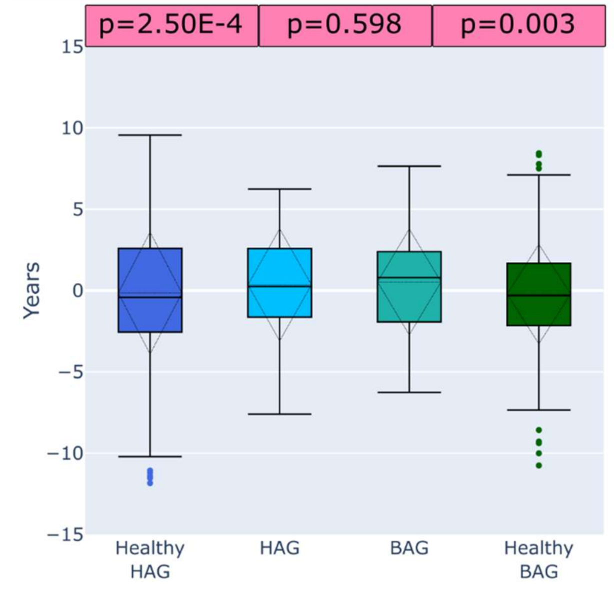

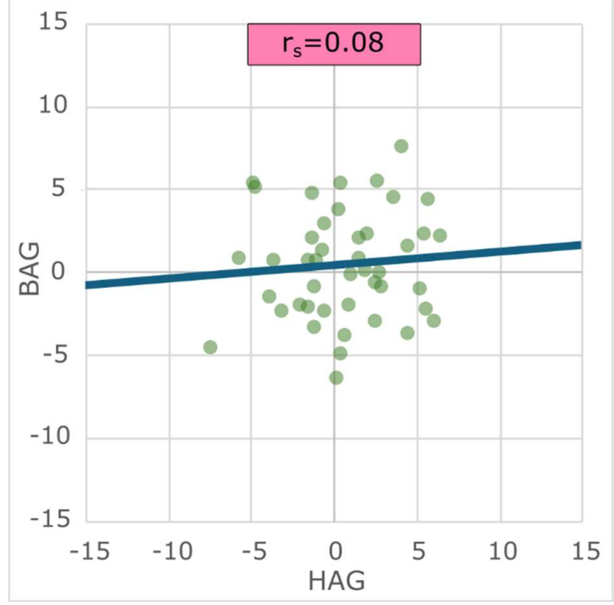

2.7. Heart valve disorders

Phecode	***ICD10 codes***
395	I35.9, I08.2, I08.1, I39, I34.0, I34.1, I35, I34.2, I34.9, I34, I36.1, I36.0, I36.2, I36.9, I36, I34.8, I36.8, Z95.4, Z95.2, Z95.3, I37.9, I37, I37.1, I37.0, I37.2

Female

BAG mean= 0.172	BAG MAE= 2.375	HAG mean= -0.083	HAG MAE= 2.306

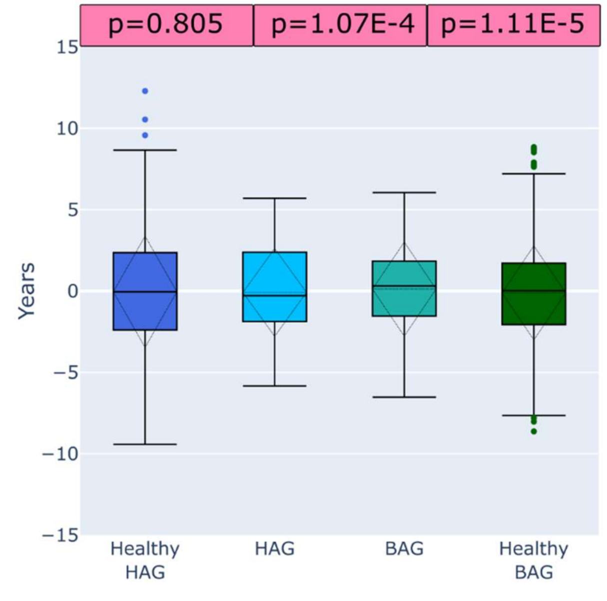

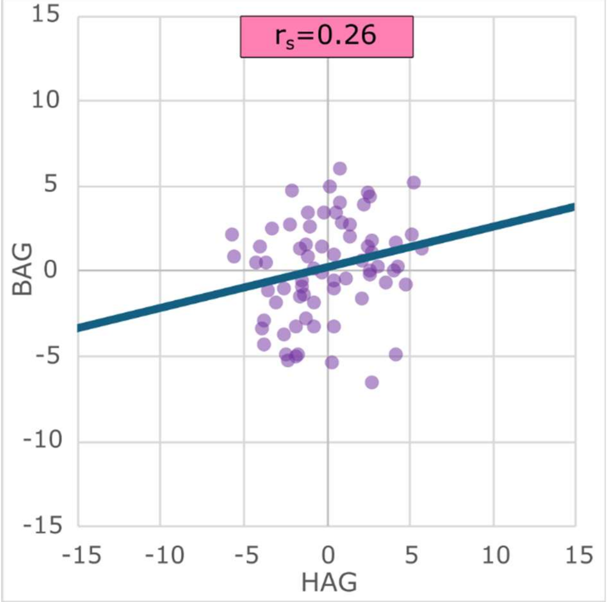

Male

BAG mean= 0.087	BAG MAE= 2.605	HAG mean= 0.047	HAG MAE= 3.345

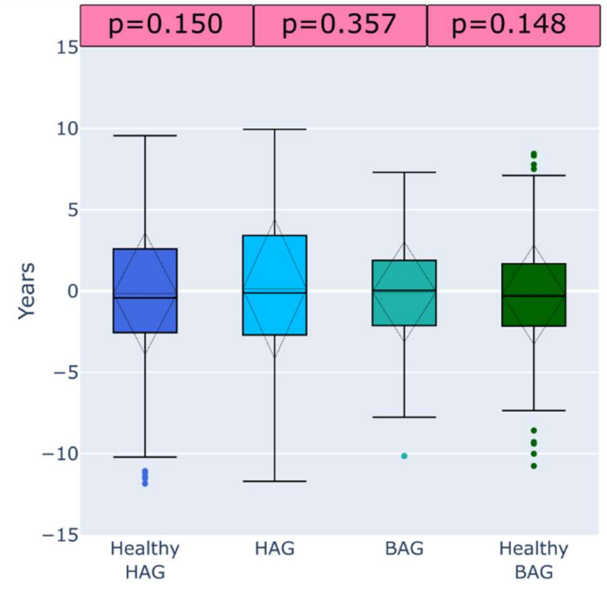

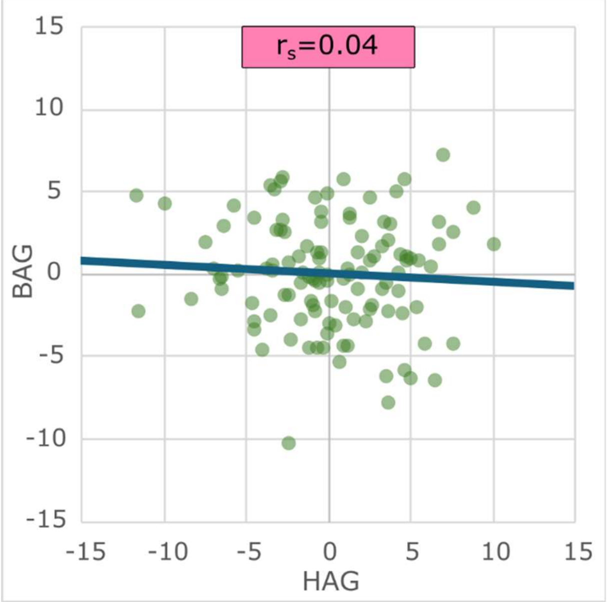

2.8. Hemorrhoids

Phecode	***ICD10 codes***
455	I84.4, I84.5, I84.3, I84, I84.1, I84.2, I84.0, I84.6, I84.8, I84.9, I84.7

Female			
BAG mean= -0.070	BAG MAE= 2.301	HAG mean= -0.048	HAG MAE= 2.926

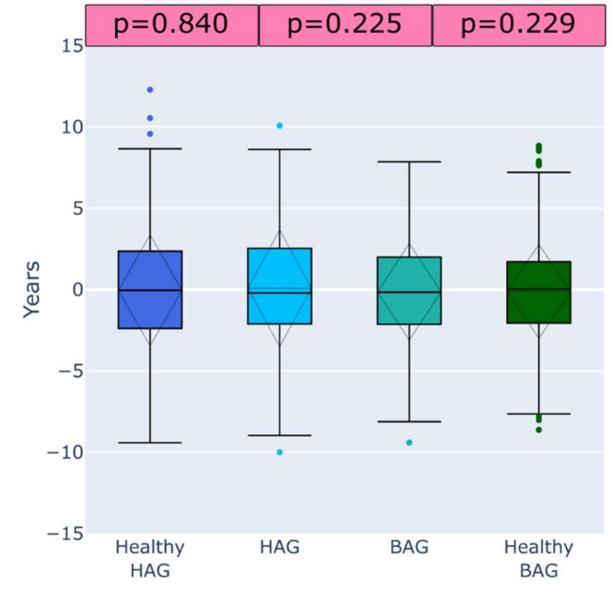

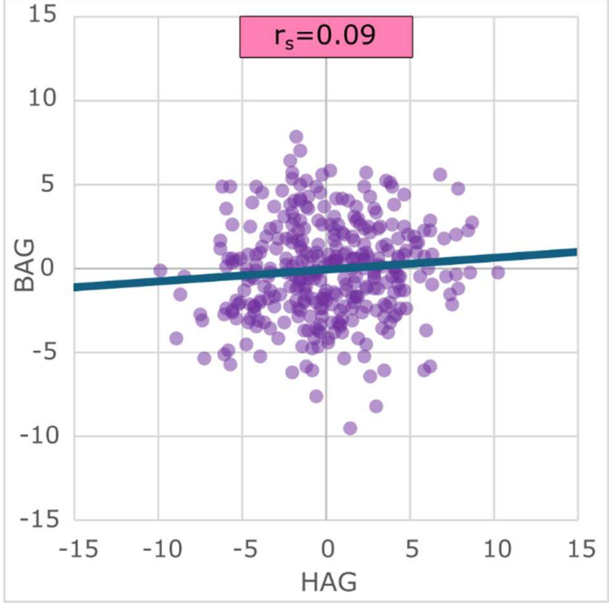

Male			
BAG mean= 0.134	BAG MAE= 2.546	HAG mean= -0.165	HAG MAE= 3.059

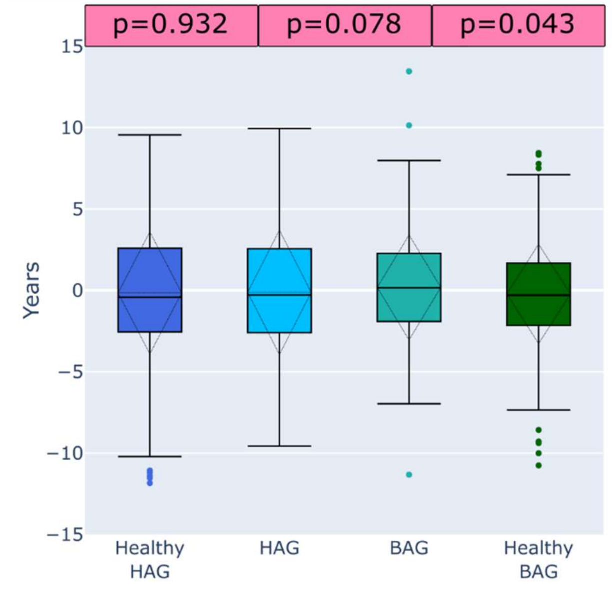

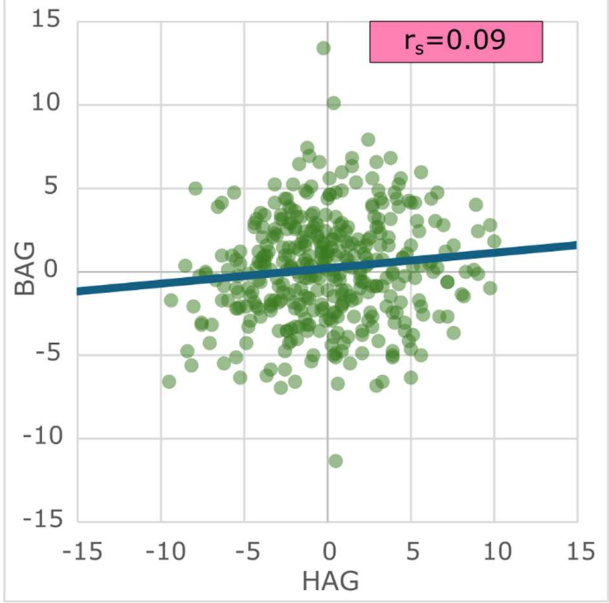

2.9. Hypertension

Phecode	***ICD10 codes***
401	I10, I15.2, I15.1, I67.4, I13, I13.0, I13.2, I13.1, I13.9, I11, I11.0, I11.9, I12, I12.0, I12.9, I15.8, I15.0, I15, I15.9

Female

BAG mean= 0.446	BAG MAE= 2.421	HAG mean= 0.520	HAG MAE= 2.701

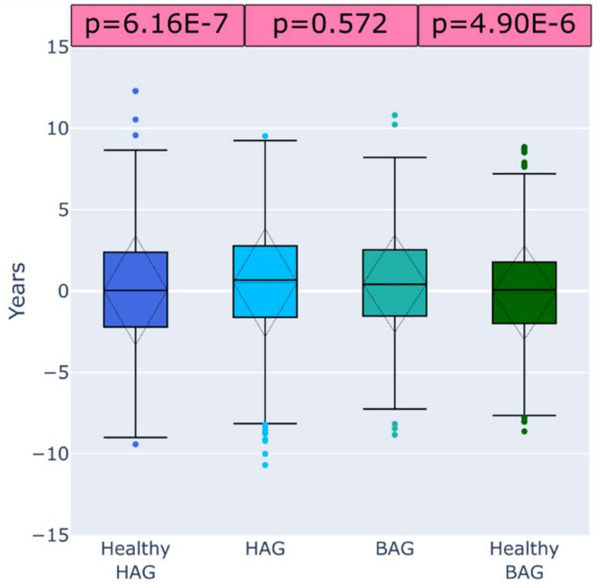

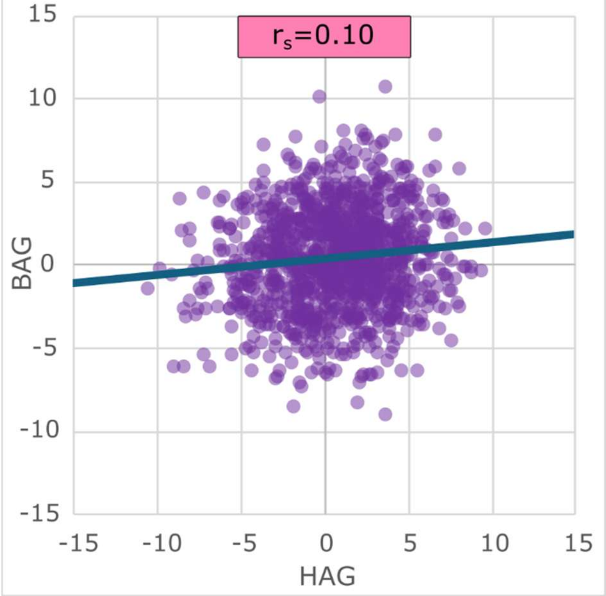

Male

BAG mean= 0.333	BAG MAE= 2.469	HAG mean= 0.265	HAG MAE= 2.964

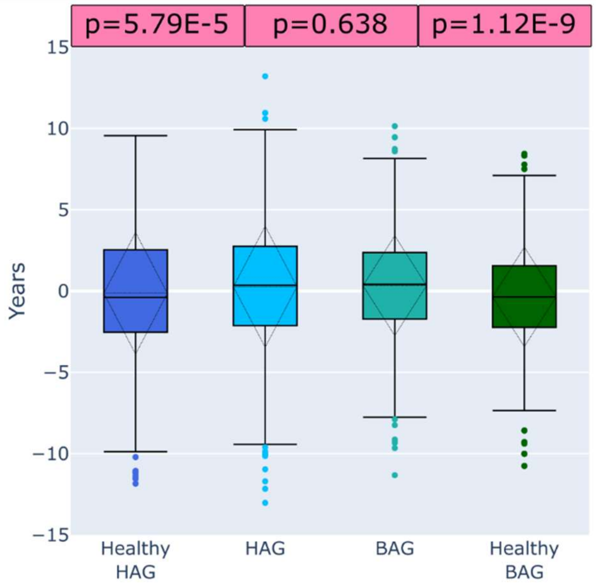

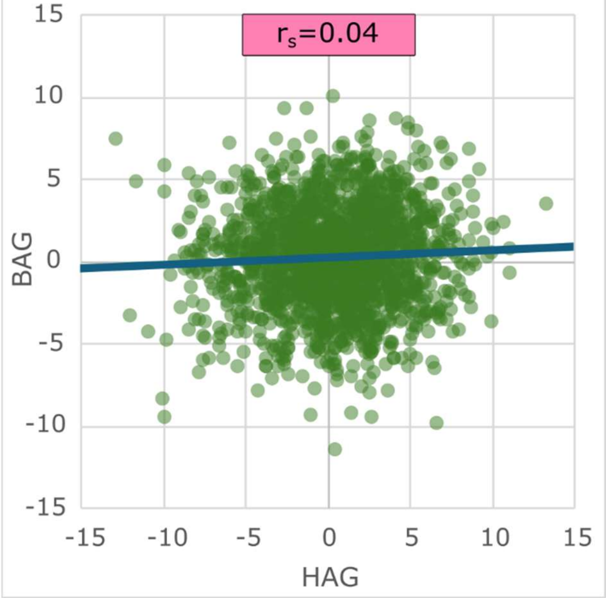

2.10. Hypotension

Phecode	***ICD10 codes***
458	I95, I95.2, I95.9, I95.0, R03.1, I95.1, I95.8

Female

BAG mean= 0.108	BAG MAE= 2.466	HAG mean= -0.856	HAG MAE= 2.998

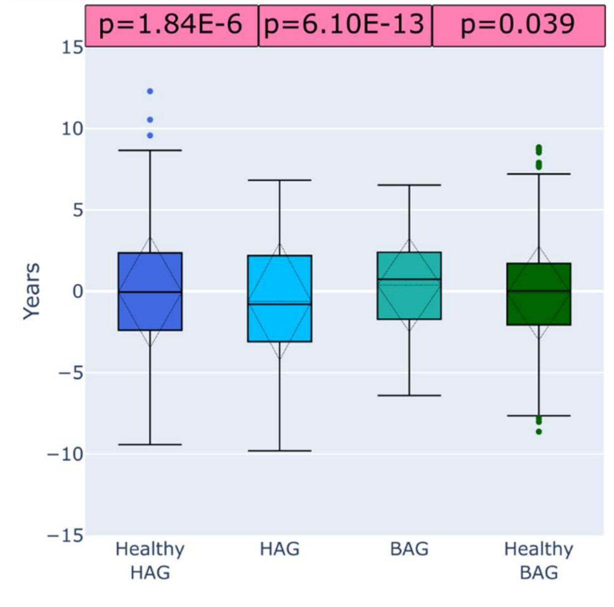

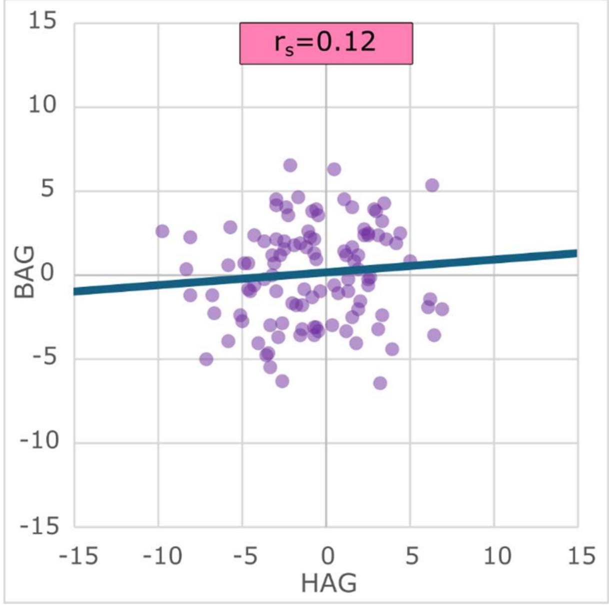

Male

BAG mean= 0.048	BAG MAE= 2.466	HAG mean= -0.058	HAG MAE= 3.082

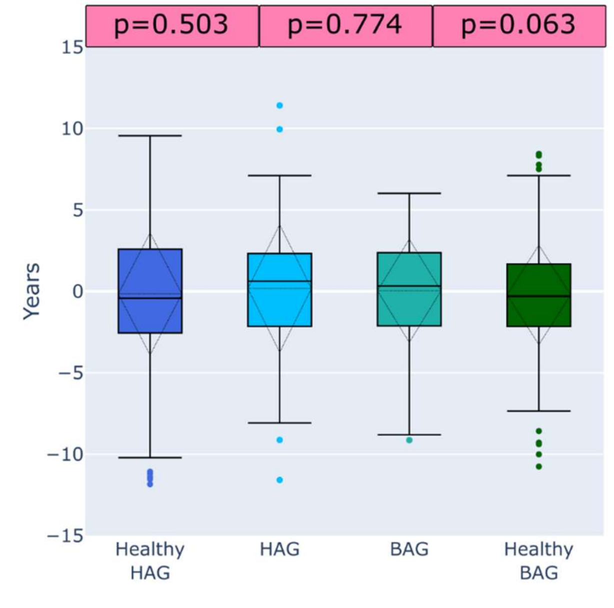

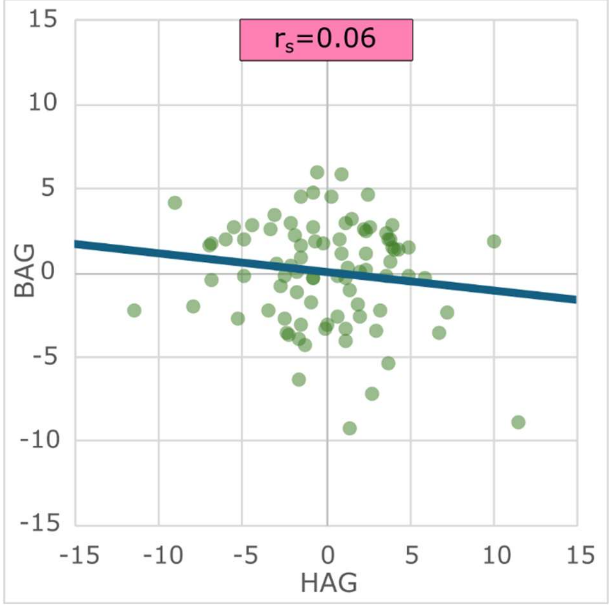

2.11. Nonspecific chest pain

Phecode	***ICD10 codes***
418	R07.4, R07.3, R07.2

Female

BAG mean= -0.501	BAG MAE= 2.459	HAG mean= -0.673	HAG MAE= 2.978

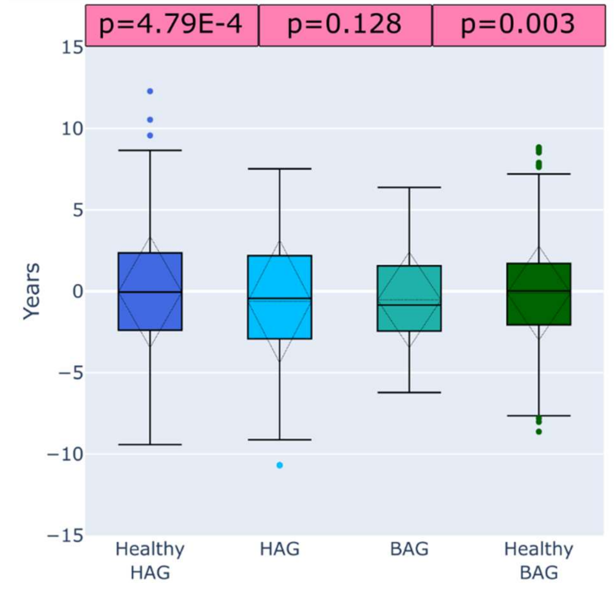

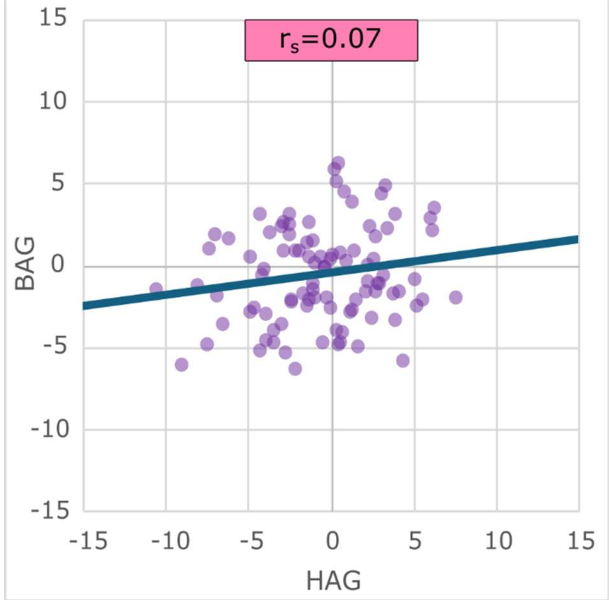

Male

BAG mean= 0.087	BAG MAE= 2.169	HAG mean= 0.117	HAG MAE= 2.770

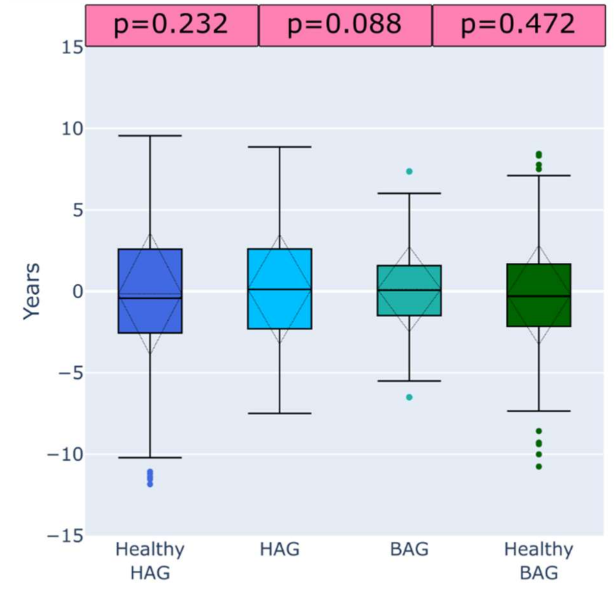

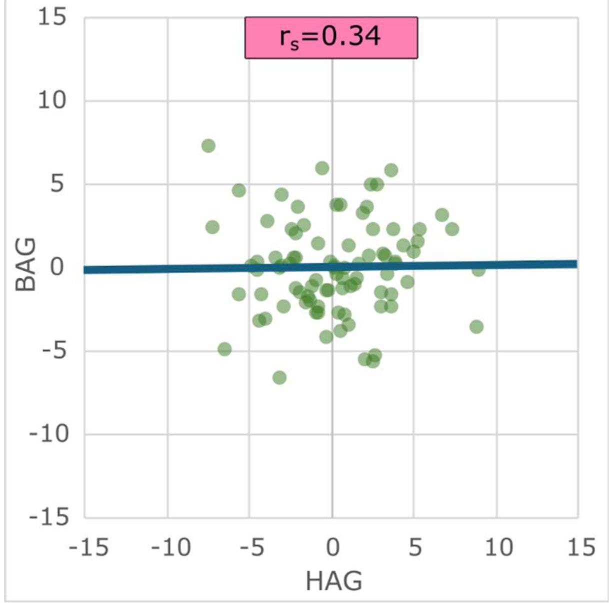

2.12. Non-cerebral aneurysm

Phecode	***ICD10 codes***
442	I71.3, I71.4, I72.4, I72.1, I72.0, I72.3, I72.8, I72.2, I72.9, I79.0, I71.8, I71.9, I71.0, I67.0, I72, I71.1, I71.2, I71.5, I71.6

Male			
BAG mean= 1.061	BAG MAE= 3.081	HAG mean= 0.686	HAG MAE= 3.980

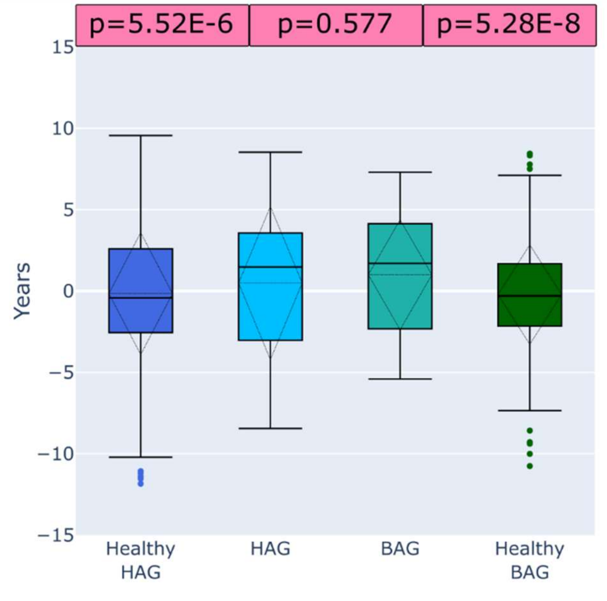

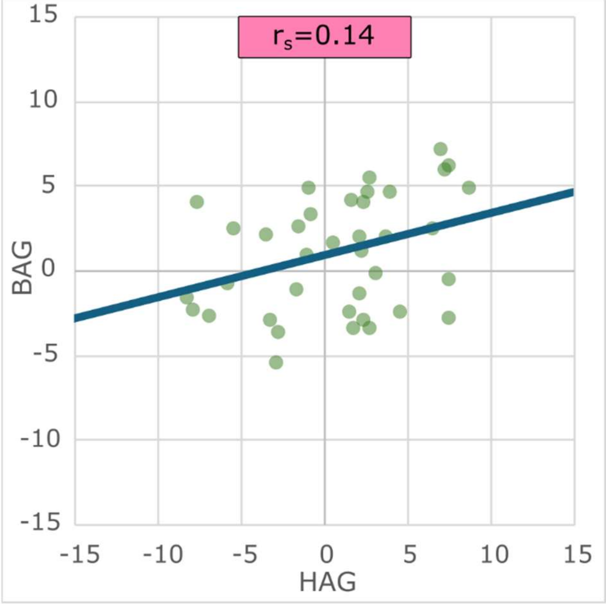

2.13. Other disorders of circulatory system

Phecode	***ICD10 codes***
459	I87.1, I87.9, R58, I99, I97.1, I87.8, Z86.7, I60.0

Female

BAG mean= -0.058	BAG MAE= 2.348	HAG mean= 0.249	HAG MAE= 2.899

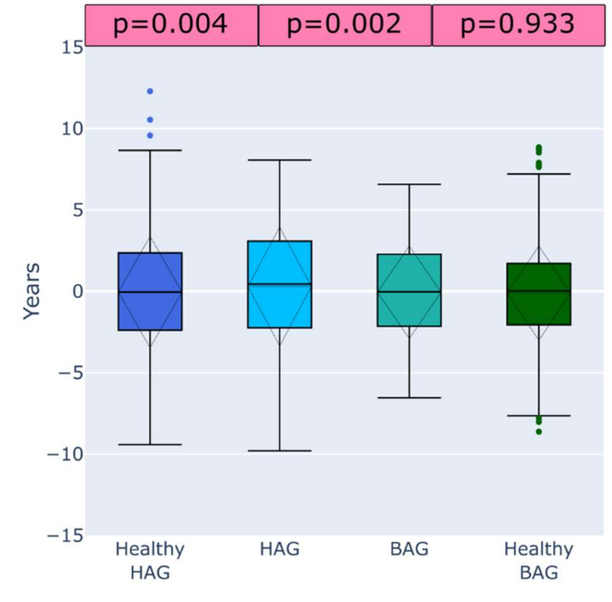

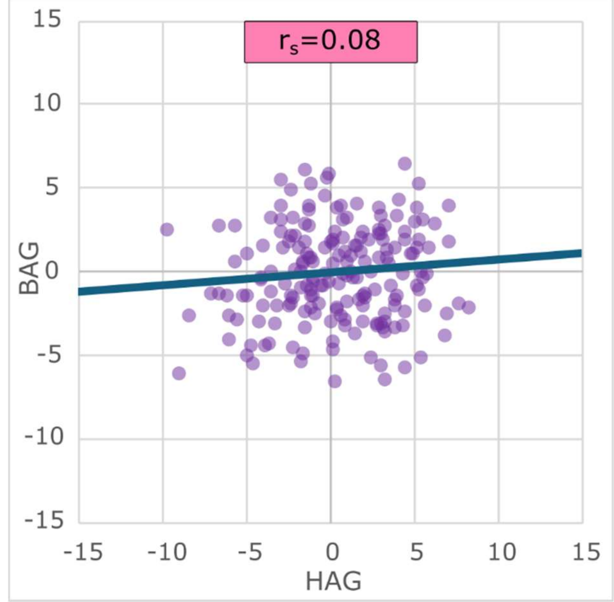

Male

BAG mean= 0.534	BAG MAE= 2.527	HAG mean= -0.130	HAG MAE= 3.042

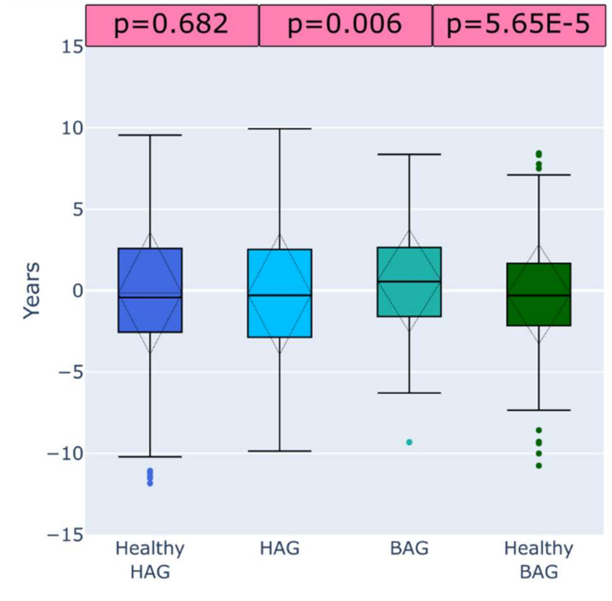

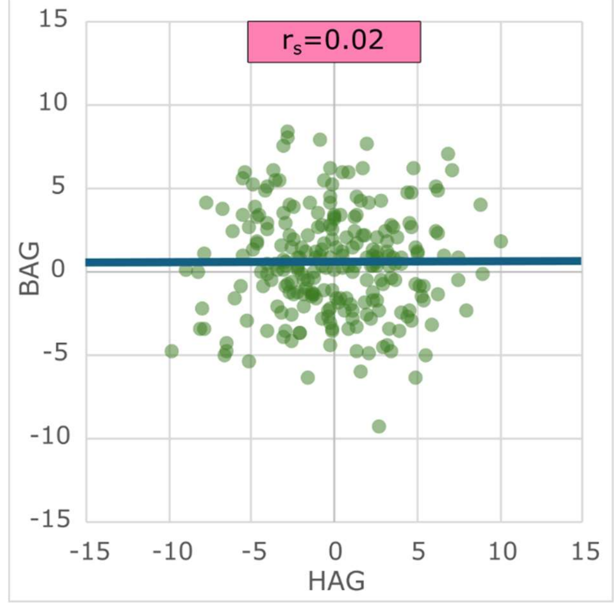

2.14. Other forms of chronic heart disease

Phecode	***ICD10 codes***
414	I51.9, I51.5, I52, I51.8, I23.4, I51.1, I23.5, I51.2, I46.1

Female

BAG mean= -0.137	BAG MAE= 2.350	HAG mean= 0.670	HAG MAE= 2.392

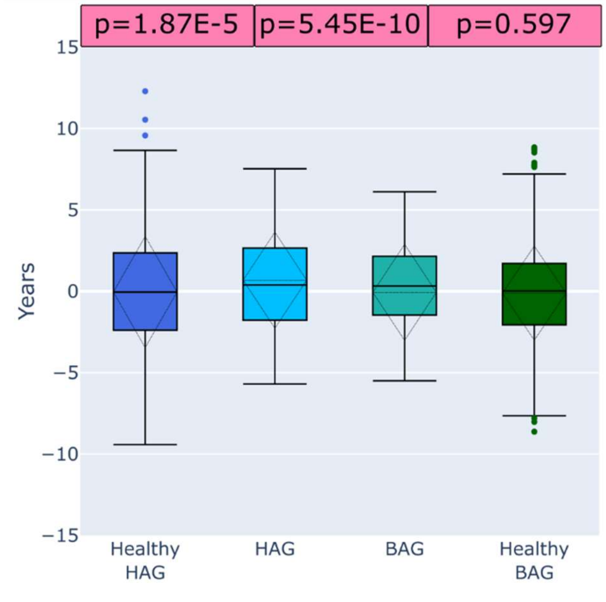

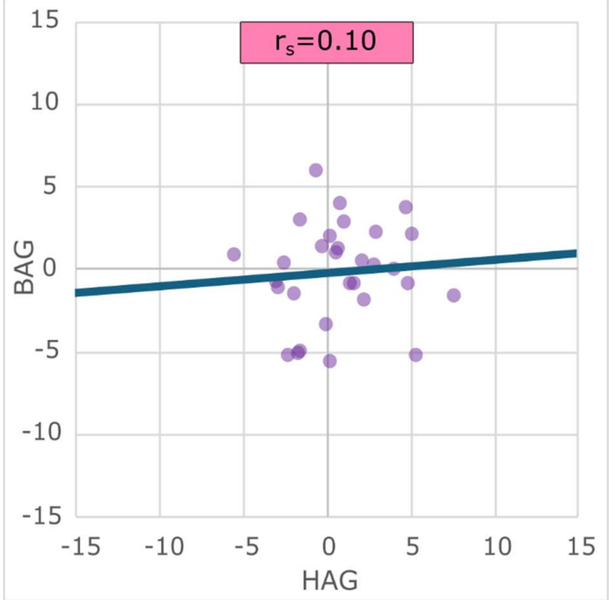

Male

BAG mean= 0.171	BAG MAE= 2.410	HAG mean= -0.539	HAG MAE= 3.162

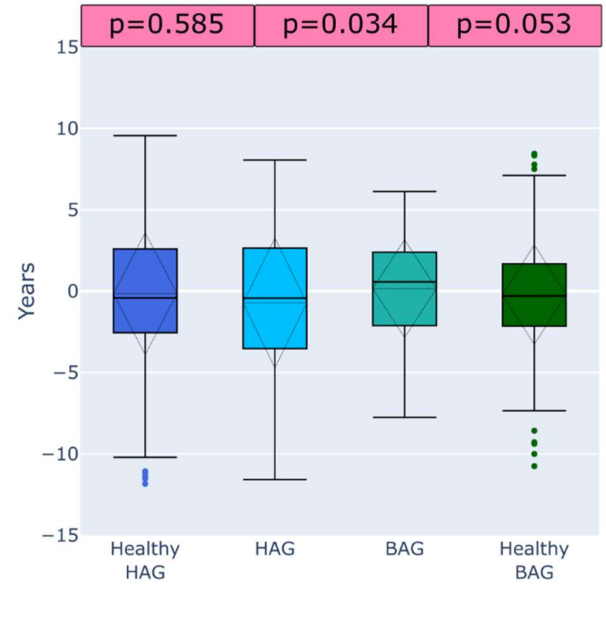

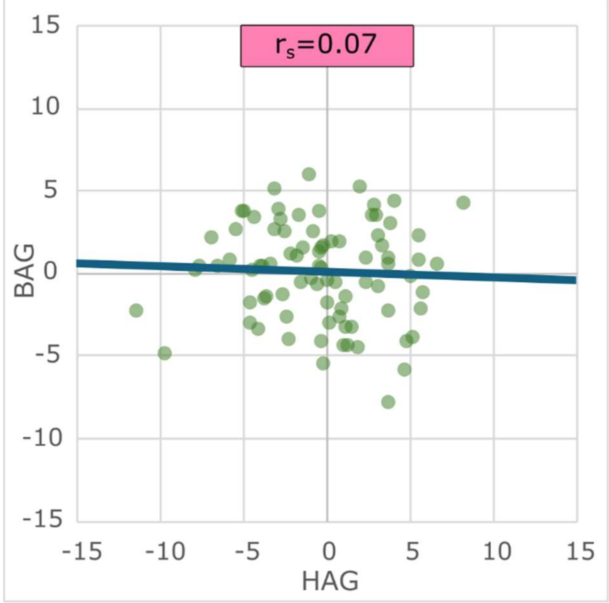

2.15. Paroxysmal tachycardia

Phecode	***ICD10 codes***
427	I48, I49.1, R00.1, I46, I46.0, I46.9, I49.9, I49.2, I49.4, I49.4, I49.8, R00.2, I47, I47.9, I47.0, I49.5, I47.1, R00.0, I49.0, I49.3, I47.2

Female

BAG mean= -0.014	BAG MAE= 2.372	HAG mean= 0.371	HAG MAE= 2.856

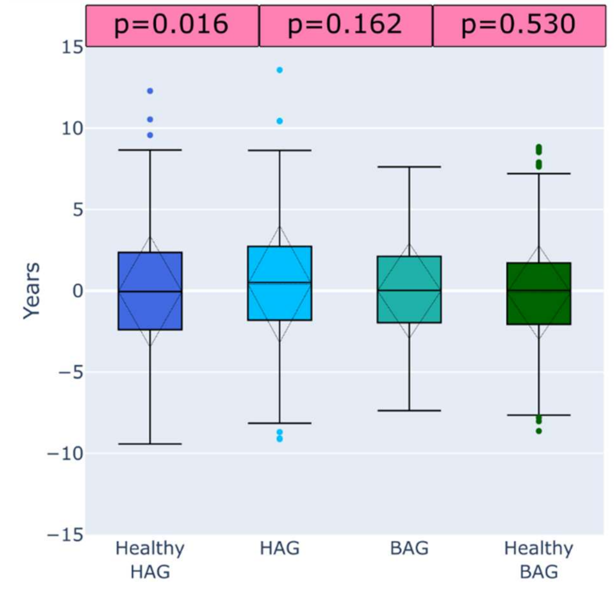

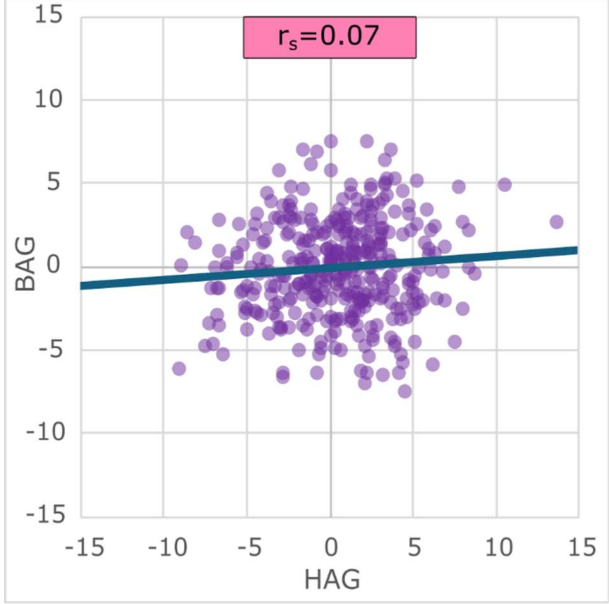

Male

BAG mean= 0.081	BAG MAE= 2.572	HAG mean= 0.260	HAG MAE= 3.156

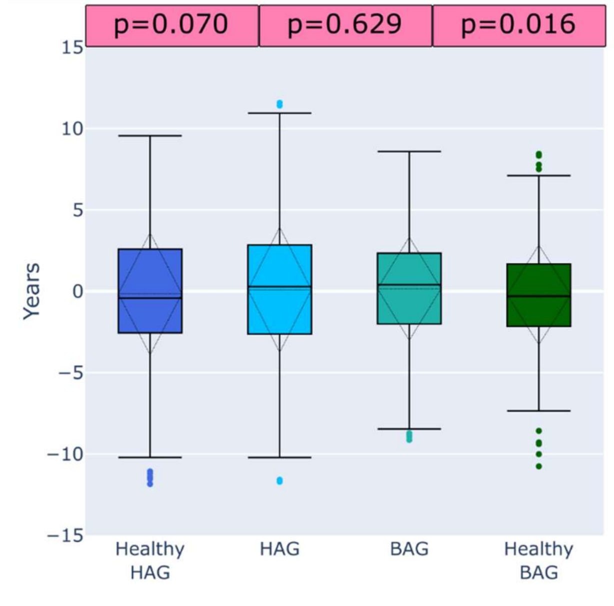

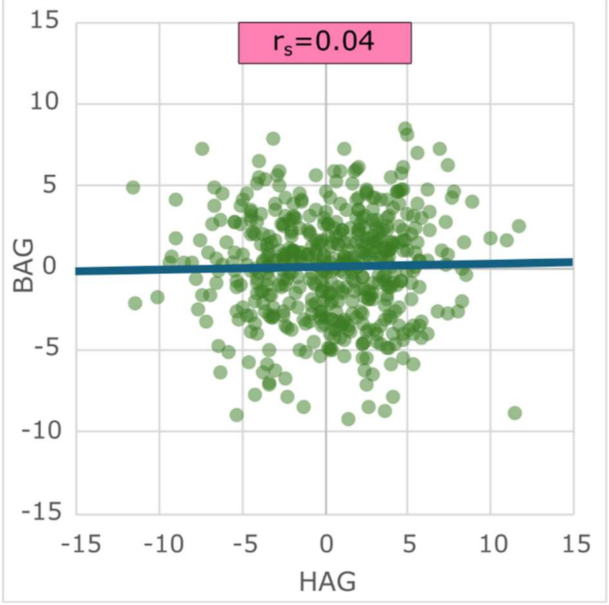

2.16. Phlebitis and thrombophlebitis

Phecode	***ICD10 codes***
451	I80, I80.1, I80.3, I80.2, I80.8, I80.0, I80.9

Male			
BAG mean= 0.906	BAG MAE= 2.709	HAG mean= 0.149	HAG MAE= 3.330

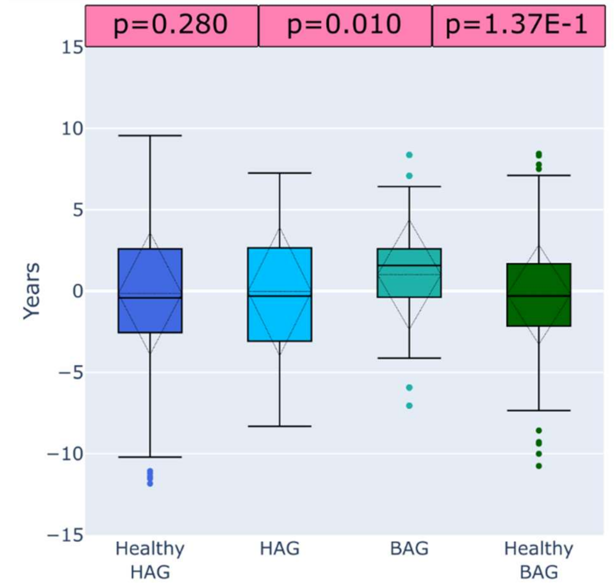

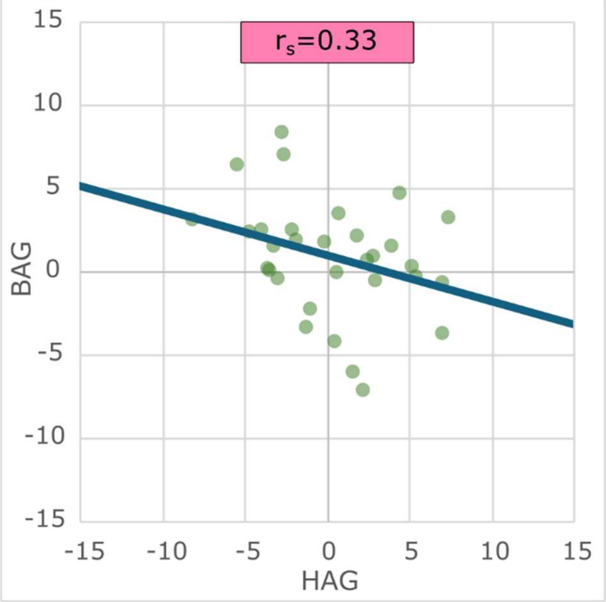

2.17. Pulmonary heart disease

Phecode	***ICD10 codes***
415	I28.1, I28.0, I28.9, I27.1, I28, I28.8, I27.8, I27.0, I26, I26.0, I26.9, I27.9

Female

BAG mean= -0.426	BAG MAE= 1.965	HAG mean= -0.568	HAG MAE= 3.563

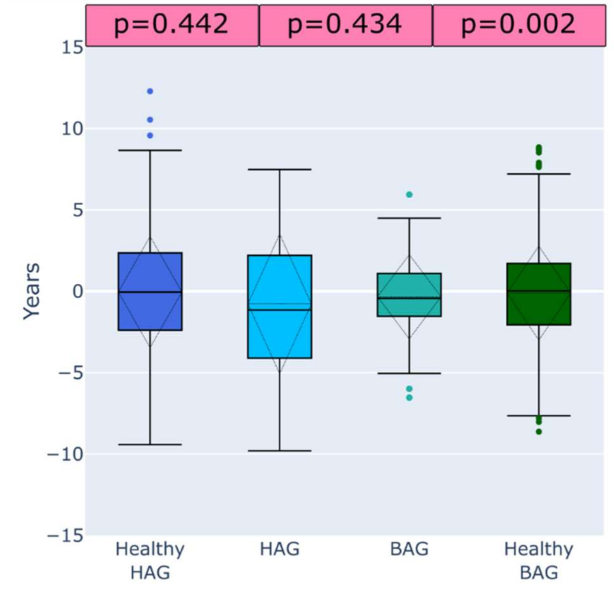

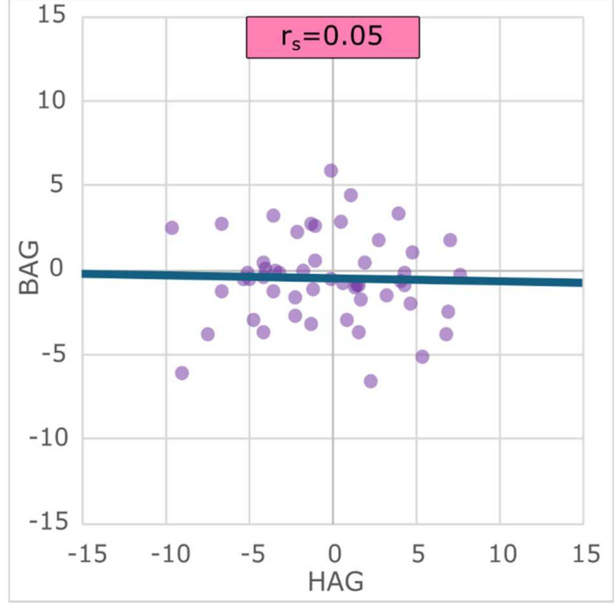

Male

BAG mean= 0.482	BAG MAE= 2.179	HAG mean= -0.105	HAG MAE= 2.551

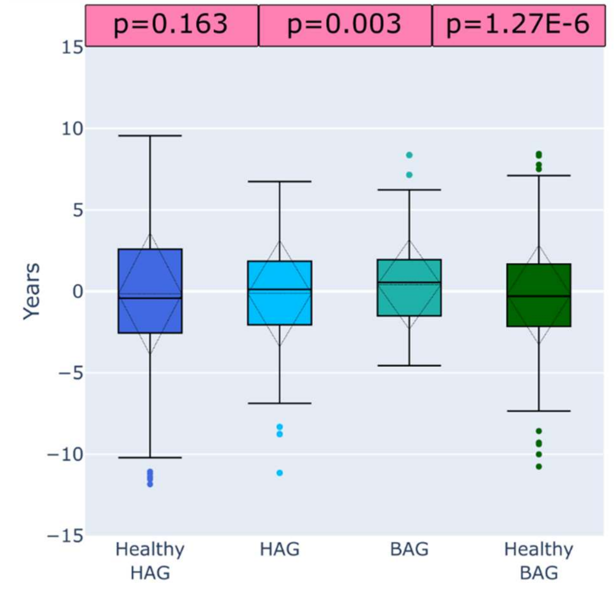

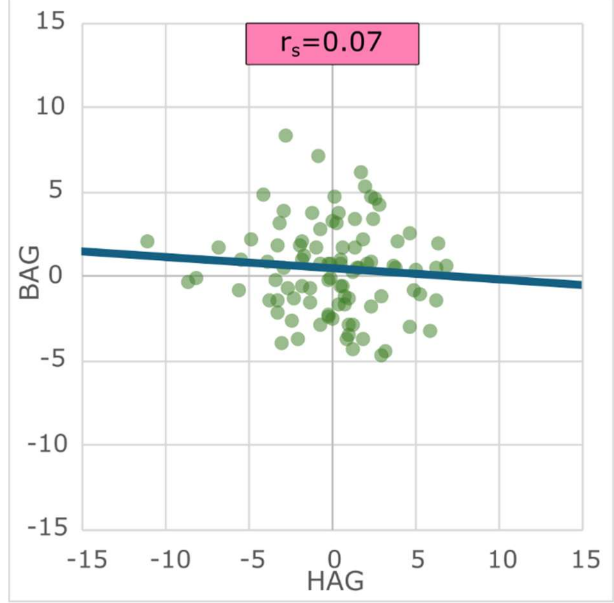

2.18. Raynaud's syndrome

Phecode	***ICD10 codes***
443	I79.1, I79.8, I73, I73.8, I79.2, I73.9, I73.0, E10.5, E11.5, E14.5

Female

BAG mean= 0.328	BAG MAE= 2.149	HAG mean= 0.138	HAG MAE= 2.996

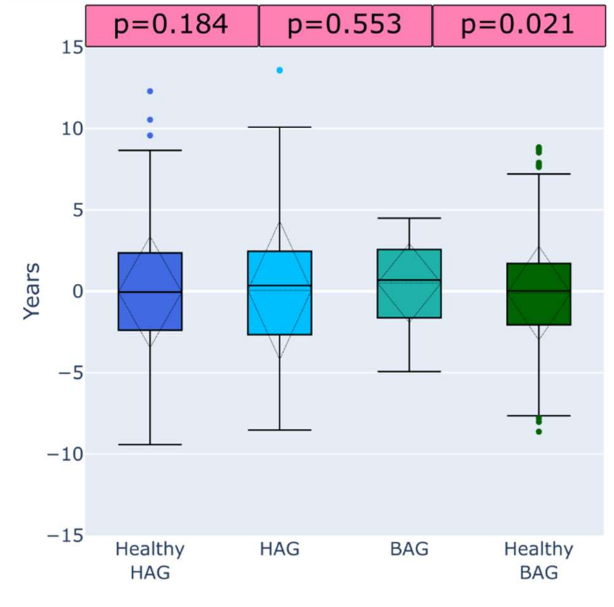

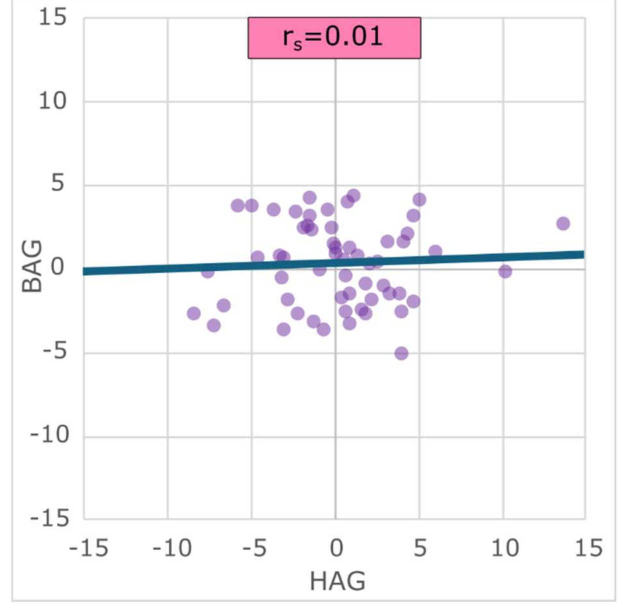

Male

BAG mean= 0.298	BAG MAE= 2.542	HAG mean= -0.031	HAG MAE= 3.380

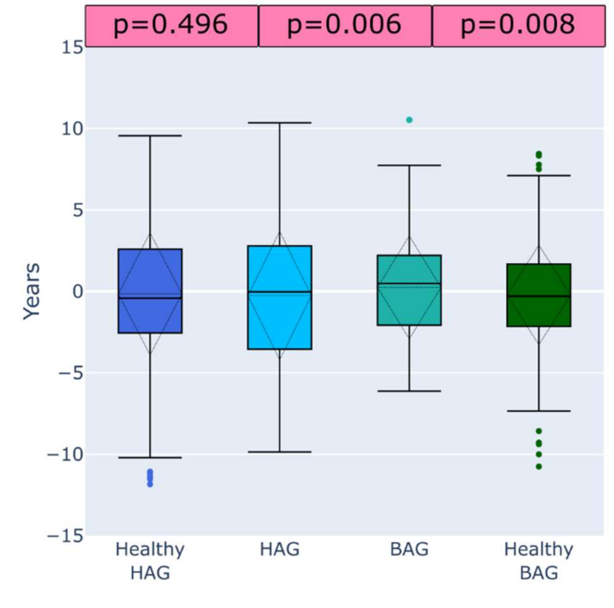

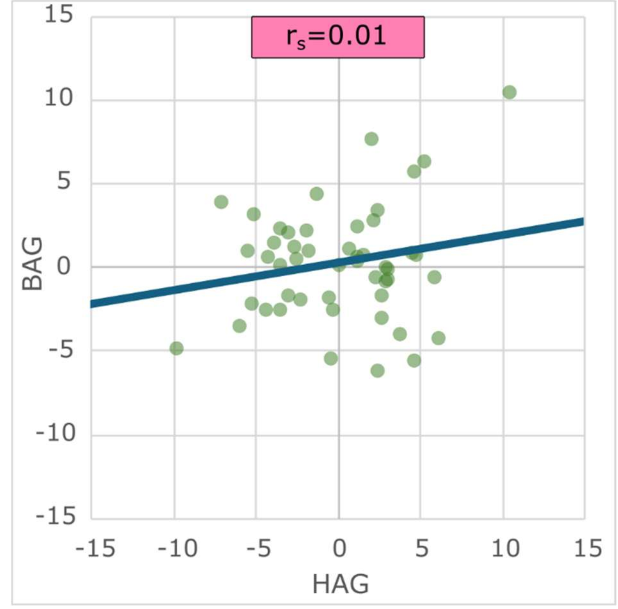

2.19. Rheumatic disease of the heart valves

Phecode	***ICD10 codes***
394	I34.1, I05.0, I05.2, I05.9, I08.9, I01.8, I35.8, I05.8, I08.8, I06.8, I09, I09.8, I07.8, I06.1, I06.0, I06.2, I06.9, I06, I02.0, I09.1, I01, I09.9, I05.1, I05, I07, I07.1, I07.0, I07.2, I07.9

Female

BAG mean= 0.437	BAG MAE= 2.369	HAG mean= 0.080	HAG MAE= 2.695

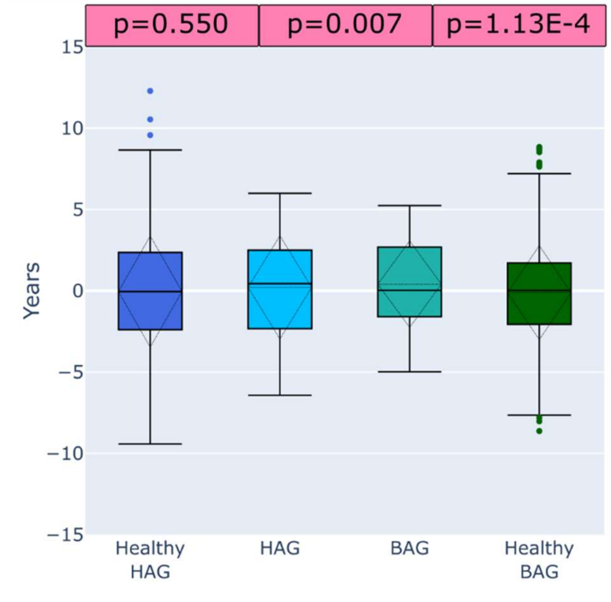

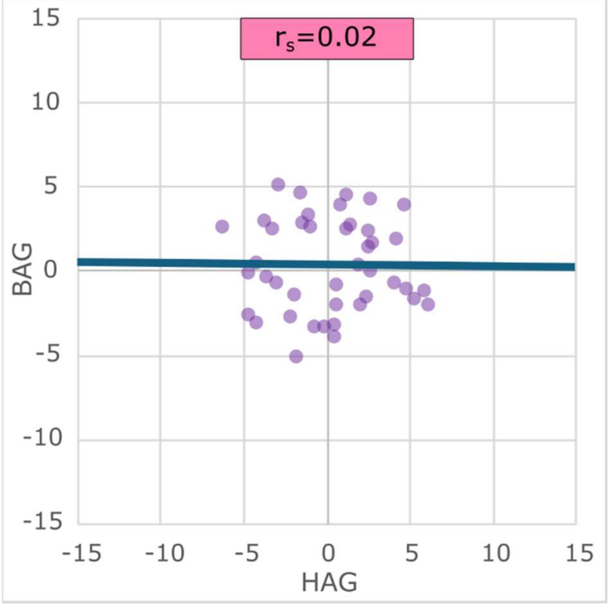

Male

BAG mean= -0.287	BAG MAE= 2.104	HAG mean= -0.876	HAG MAE= 3.546

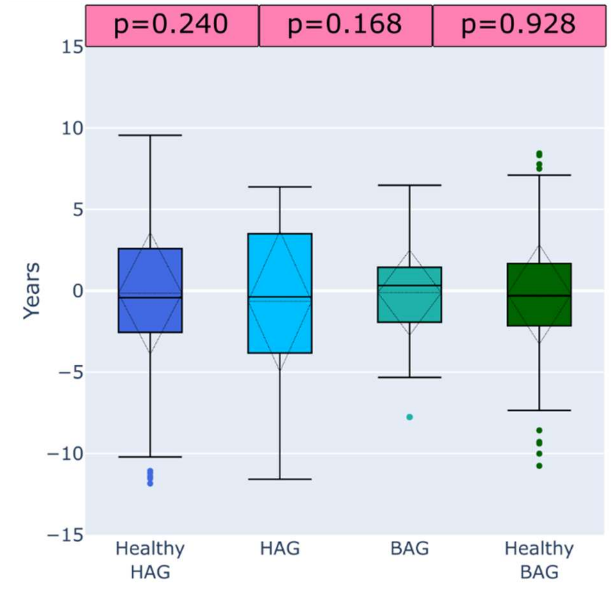

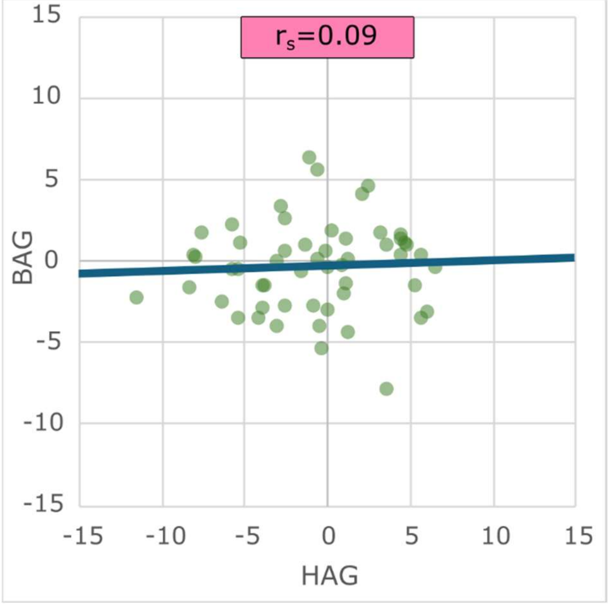

2.20. Unstable angina intermediate coronary syndrome

Phecode	***ICD10 codes***
411	I51.0, I23, I25, I25.9, I25.4, I24.0, I24.1, I23.0, I51.3, I25.5, I34.1, I25.2, I24, I23.8, I24.8, I20.8, I25.8, Z95.1, Z95.5, I23.3, I25.6, I22, I22.0, I22.1, I22.8, I22.9, I23.6, I20.0, I23.2

Female

BAG mean= -0.116	BAG MAE= 2.388	HAG mean= 0.039	HAG MAE= 2.906

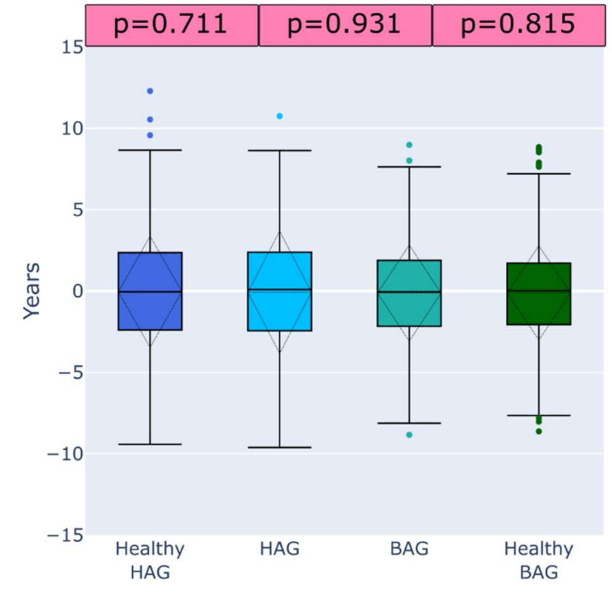

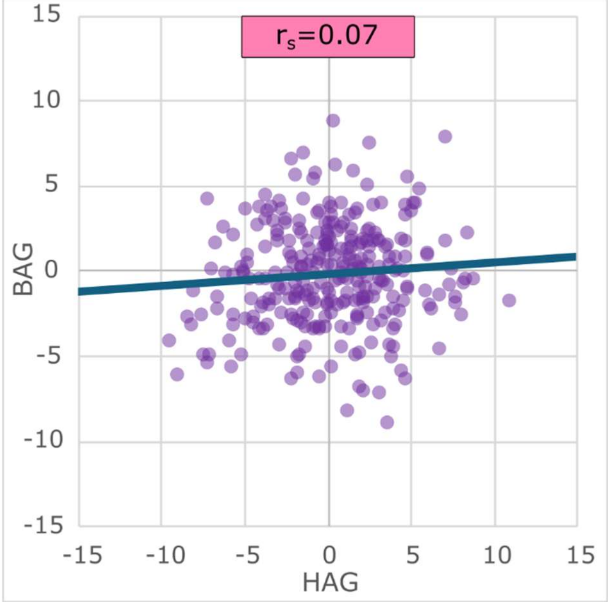

Male

BAG mean= 0.280	BAG MAE= 2.449	HAG mean= 0.092	HAG MAE= 3.035

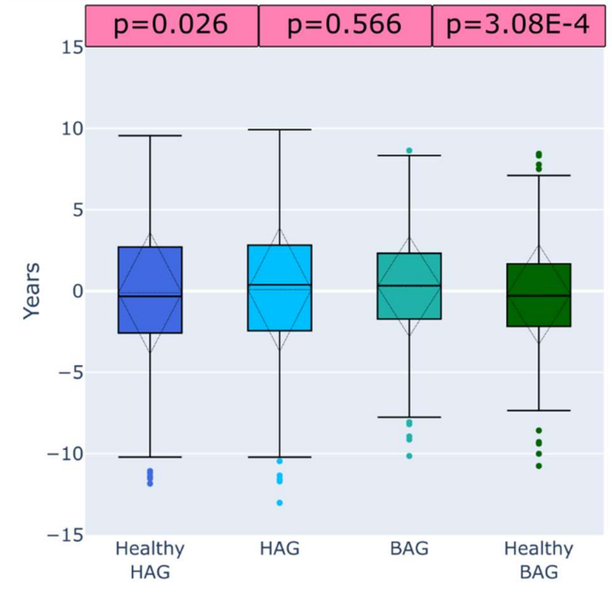

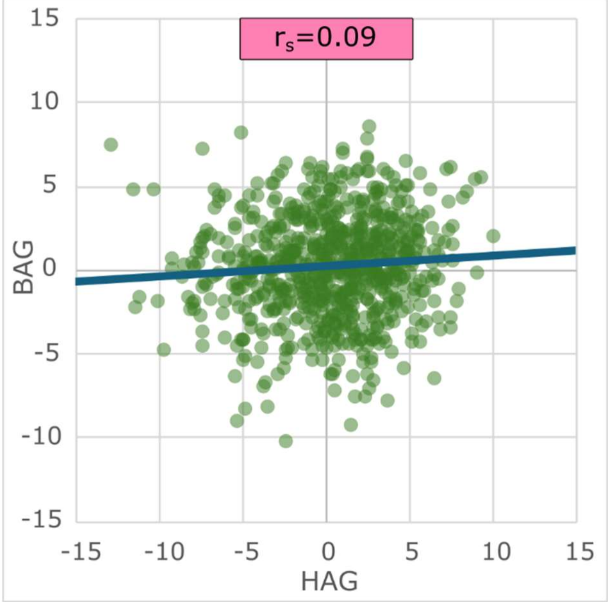

2.21. Varicose veins

<table>
<tr><th>Phecode</th><th colspan="3">ICD10 codes</th></tr>
<tr><td>454</td><td colspan="3">I86.4, I86.2, I86.1, I86.0, I83, I83.2, I83.1, I83.0, I83.9, I86, I86.8, I86.3</td></tr>
<tr><td colspan="4">Female</td></tr>
<tr><td>BAG mean= -0.091</td><td>BAG MAE= 2.196</td><td>HAG mean= -0.251</td><td>HAG MAE= 2.805</td></tr>
<tr><td colspan="2">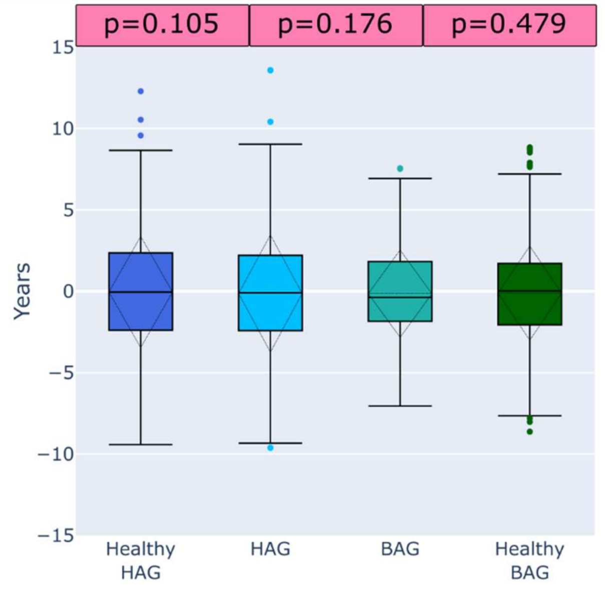
</td><td colspan="2">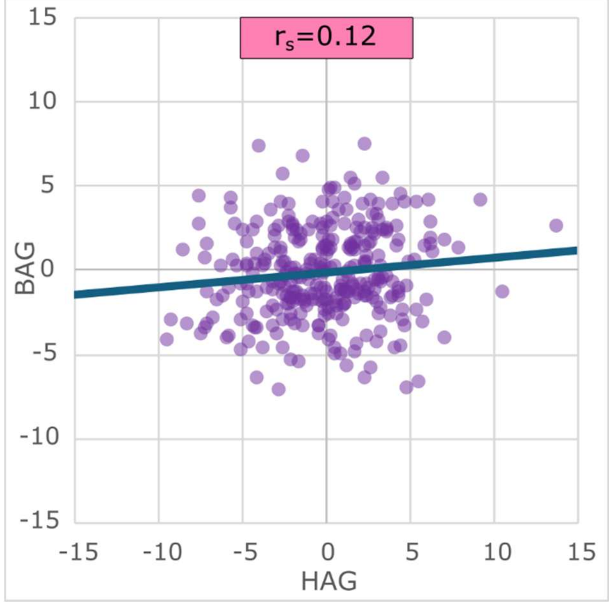
</td></tr>
<tr><td colspan="4">Male</td></tr>
<tr><td>BAG mean= 0.325</td><td>BAG MAE= 2.545</td><td>HAG mean= -0.610</td><td>HAG MAE= 2.962</td></tr>
<tr><td colspan="2">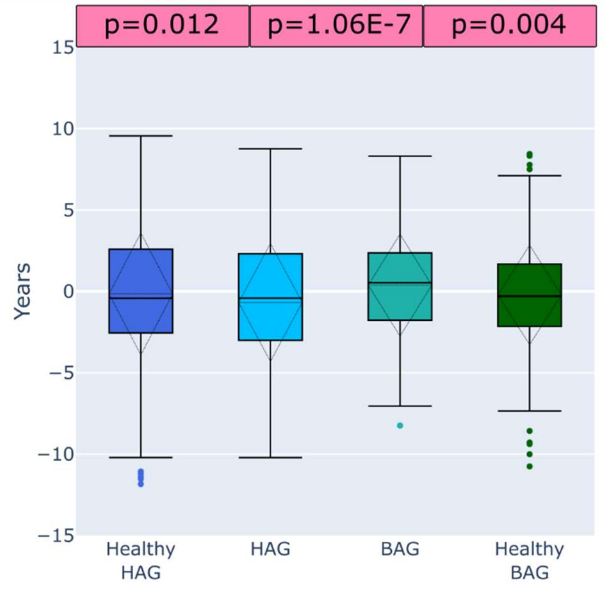
</td><td colspan="2">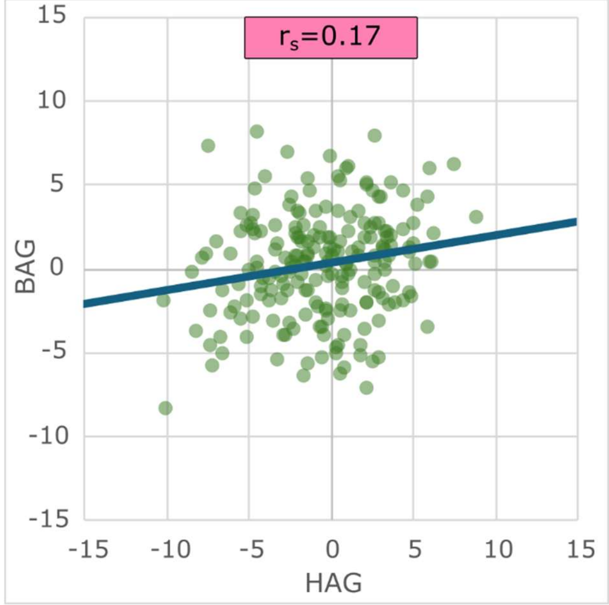
</td></tr>
</table>
